# Regenerative Endodontic Therapies: Harnessing Stem Cells, Scaffolds, and Growth Factors

**DOI:** 10.3390/polym17111475

**Published:** 2025-05-26

**Authors:** Rosana Farjaminejad, Samira Farjaminejad, Franklin Garcia-Godoy

**Affiliations:** 1 Department of Health Services Research and Management, School of Health and Psychological Sciences, City, University of London, London WC1E 7HU, UK; samira.farjaminejad@city.ac.uk; 2 Department of Bioscience Research, Bioscience Research Center, College of Dentistry, University of Tennessee Health Science Center, 875 Union Avenue, Memphis, TN 38163, USA; godoy@uthsc.edu

**Keywords:** dental, regeneration, dental pulp stem cells, collagen, transforming growth factor beta, 3D bioprinting

## Abstract

Regenerative Endodontic Therapies (RETs) offer transformative potential by leveraging polymer-based scaffolds, stem cells, and growth factors to regenerate damaged dental pulp tissue, thereby restoring tooth vitality and prolonging tooth function. While conventional treatments focus on infection control, they often compromise the structural and biological integrity of the tooth. RETs, in contrast, aim to restore the natural function of the pulp–dentin complex by promoting cellular regeneration and immune modulation. In this context, biodegradable polymers—such as collagen, gelatin methacryloyl (GelMA), and synthetic alternatives—serve as scaffolding materials that mimic the extracellular matrix, support cell attachment and proliferation, and enable localized delivery of bioactive factors. Together, the tissue engineering triad—polymer-based scaffolds, stem cells, and signaling molecules—facilitates root development, apical closure, and increased fracture resistance. Recent innovations in polymeric scaffold design, including injectable hydrogels and 3D bioprinting technologies, have enhanced clinical translation by enabling minimally invasive and patient-specific RETs. Despite progress, challenges such as immune compatibility, scaffold degradation rates, and the standardization of clinical protocols remain. RETs, thus, represent a paradigm shift in dental care, aligning with the body’s intrinsic healing capacity and offering improved long-term outcomes for patients.

## 1. Introduction

**Figure 1:**
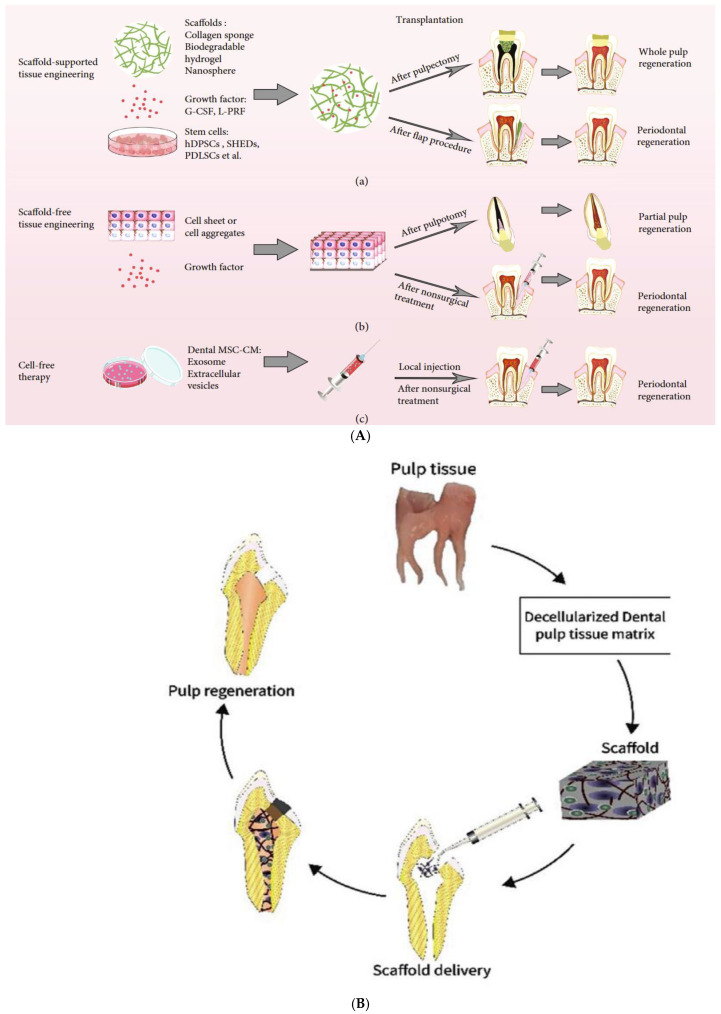
(**A**) Three therapeutic strategies have been proposed for treating endodontic and periodontal diseases using dental mesenchymal stem cells (MSCs): (**a**) dental tissue regeneration through the classic tissue engineering model, which involves the use of dental MSCs combined with supporting biomaterial scaffolds and growth factors; (**b**) dental tissue regeneration via scaffold-free tissue engineering approaches; and (**c**) a cell-free strategy that promotes dental tissue regeneration using conditioned medium (CM) containing exosomes and/or extracellular vesicles (EVs) secreted by dental MSCs. (**B**) Pulp regeneration process using decellularized dental pulp tissue matrix. The cycle starts from the decellularization of pulp tissue to form a dental pulp tissue matrix scaffold, which is then delivered into the tooth structure for regeneration. Adopted from [10,12].

Regenerative Endodontic Therapies (RETs) aim to transform the management of dental pulp necrosis by leveraging the body’s intrinsic regenerative abilities [1]. Traditional endodontic treatments focus on removing diseased pulp and filling the canal with inert materials to prevent reinfection. While effective at preserving tooth structure, these methods fail to restore critical biological functions of the pulp, such as immune defense, sensory perception, and dentin production [2]. In contrast, RETs seek to regenerate the pulp–dentin complex, restoring natural tooth function and extending the longevity of treated teeth through the application of stem cells, scaffolds, and growth factors (Figure 1) [3].

RETs offer a promising alternative to conventional approaches like apexification, which merely closes the root apex without enabling further root development. By replacing necrotic pulp with functional tissue, RETs promote root lengthening, the thickening of dentin walls, and overall strengthening of the tooth, significantly reducing fracture risk. A key component of this approach is the RET triad: stem cells, growth factors, and scaffolds. Stem cells from the apical papilla (SCAP), for instance, have demonstrated the ability to differentiate into odontoblast-like cells that support dentin formation and mimic natural tooth development [4,5,6].

The biological foundation of RETs lies in the natural regenerative capacity of the pulp–dentin complex, an integrated unit capable of responding to injury with cellular and molecular repair mechanisms. Upon trauma or decay, growth factors within the root canal environment activate to guide cell differentiation and tissue organization, while scaffolds provide essential structural support for new pulp-like tissue formation [7]. RETs have consistently achieved root thickening, apical closure, and root lengthening, outperforming apexification, which often leaves roots thin and susceptible to fracture. Moreover, RETs restore critical immunologic and sensory functions, representing a significant advancement in endodontic care, particularly for young patients, where long-term tooth viability is essential.

Early RET methods involved simple scaffolds or the direct application of growth factors. Advances in cellular biology and materials science have since enabled more sophisticated techniques, including stem cell therapy, scaffold implantation, and gene therapy, each contributing uniquely to dental tissue regeneration [8]. Postnatal stem cells from dental pulp have shown great potential in clinical applications, effectively regenerating the pulp–dentin complex to restore tooth vitality and function. Injectable scaffolds, both natural and synthetic, now provide minimally invasive options, while three-dimensional cell printing and gene therapy innovations are paving the way for highly personalized regenerative treatments. Three feasible regenerative strategies based on MSCs have been proposed for clinical treatment of dental diseases [9,10,11,12] (Figure 1).

These advancements underscore the transformative potential of RETs in revolutionizing endodontic therapy, offering a biologically driven solution that aligns with the natural repair mechanisms of the tooth while significantly improving long-term outcomes.

Recent interdisciplinary studies have further highlighted the importance of optimizing experimental designs and understanding biological mechanisms in regenerative therapies [13].

**Figure 2:**
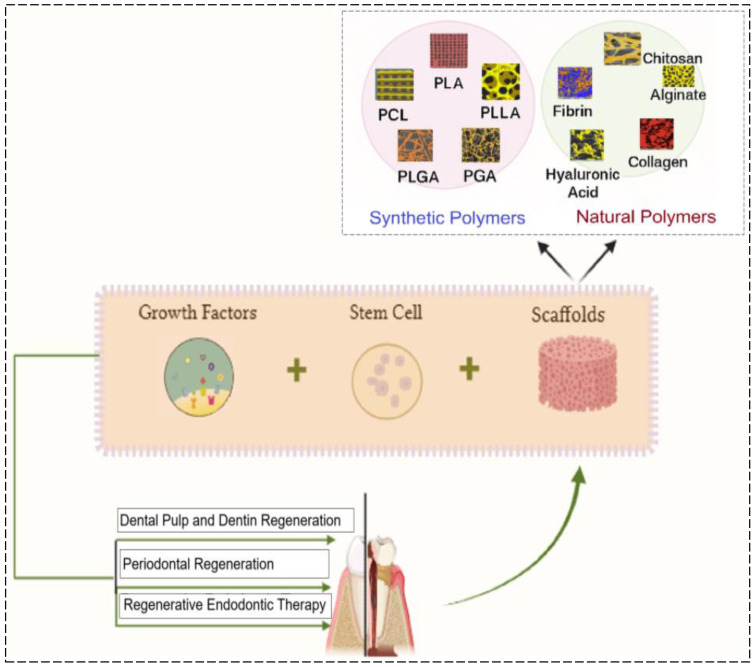
Schematic illustrating the integration of stem cells, scaffolds, and growth factors in RE for pulp–dentin regeneration. Depiction of various scaffolds used in dental TE, including natural and synthetic polymers, that support dental pulp regeneration by interacting with stem cells and growth factors.

By preserving the natural tooth structure and restoring function, RETs significantly improve patient outcomes for conditions such as pulp necrosis and apical periodontitis while also enhancing overall quality of life. As these innovative methods evolve, they hold the potential to redefine endodontic care, transitioning from traditional practices to biologically inspired approaches that harness and support the body’s innate healing capabilities [14,15] (Figure 2).

Regenerative Endodontic Therapies (RETs) represent a major shift in dental treatment, moving beyond conventional approaches that focus solely on infection control to strategies that restore tooth vitality and function. While traditional endodontic treatments are effective at eliminating infections, they often compromise tooth structure and fail to re-establish critical biological functions, such as immune defense, sensory perception, and dentin production [5,6]. This limitation is particularly significant for young patients with immature teeth, where maintaining structural integrity and biological activity is crucial for long-term tooth viability.

RETs offer biologically driven solutions by employing advanced techniques such as stem cell therapy, scaffold implantation, and bioactive molecule delivery to regenerate the damaged pulp–dentin complex. By addressing the underlying causes of tissue damage, RETs promote healing, enhance root development, and significantly strengthen the tooth structure, reducing the risk of fracture. Furthermore, RETs restore vital immune and sensory functions, contributing to long-term tooth preservation and improved patient quality of life [4].

RET has shown promising results in adults, particularly for cases involving necrotic pulp and apical pathologies [16].

Clinical applications include apical revascularization, chemo-mechanical debridement, and the use of bioactive materials such as mineral trioxide aggregate (MTA) and collagen membranes. Case studies have highlighted RETs’ versatility and adaptability in resolving symptoms, promoting root maturation, achieving apical closure, and restoring pulp vitality even under challenging conditions. Table 1 summarizes representative clinical cases, emphasizing the effectiveness of RETs across a diverse patient population.

Despite these promising results, further research and the establishment of standardized protocols remain essential to optimize outcomes, particularly in adult patients, where regenerative capacity may be reduced compared to younger individuals.

## 2. Techniques for Tissue Engineering in RETs

RETs incorporate advanced techniques designed to revitalize necrotic or damaged pulp tissue within the root canal. Key approaches include root canal revascularization, utilizing blood clots to stimulate tissue growth and repair within the disinfected root canal; postnatal stem cell therapy, introducing adult stem cells, such as those from the apical papilla, to promote regeneration of the pulp–dentin complex; and scaffold-based techniques, employing scaffold implantation or injectable hydrogels to provide structural support and a conducive environment for cell proliferation and tissue regeneration (Table 2). Emerging technologies, such as three-dimensional cell printing and gene therapy, offer significant potential for precisely recreating or enhancing pulp tissue, although these methods remain in the experimental stage [31]. These techniques collectively highlight the innovative strides being made in RETs, paving the way for more effective and personalized treatments.

### 2.1. Root Canal Revascularization via Blood Clotting

Root canal revascularization is a clinically applied RET primarily used in immature permanent teeth with necrotic pulps. The technique harnesses the patient’s own blood clot as a natural scaffold to promote tissue regeneration within the canal space [44].

The procedure starts with thorough canal disinfection using irrigants, such as sodium hypochlorite, and intracanal medicaments, like calcium hydroxide or triple-antibiotic paste, to eliminate microorganisms [45]. Once the canal is adequately disinfected, intentional over-instrumentation beyond the apical foramen is performed to initiate bleeding from the periapical tissues. The induced bleeding results in clot formation within the canal, which facilitates the ingrowth of mesenchymal stem cells and growth factors that support tissue regeneration [45].

A biocompatible matrix may be placed over the clot, followed by sealing the coronal access with materials such as MTA or bioceramic cements to prevent reinfection [46]. This technique has demonstrated clinical benefits, including continued root development, apical closure, and thickening of dentin walls, especially in young patients with developing teeth [44,46].

**Figure 3:**
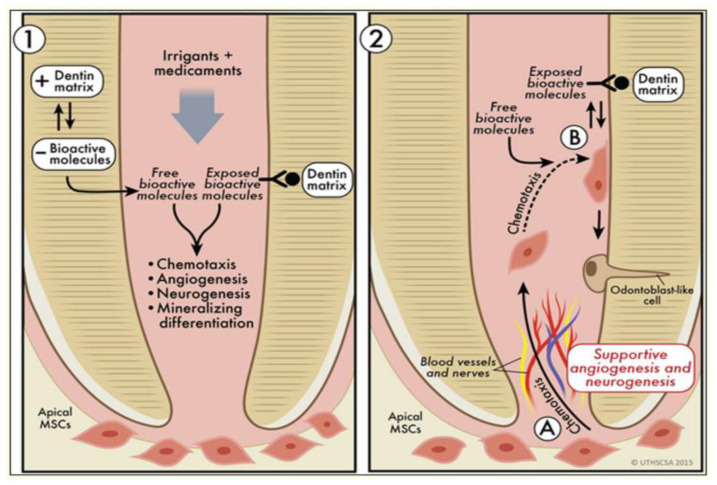
The application of irrigates and medicaments to a dentin matrix releases bioactive molecules, enhancing chemotaxis, angiogenesis, neurogenesis, and differentiation, which together stimulate odontoblasts and support regeneration within the dentin matrix. Adopted from [47].

However, variability in clinical outcomes persists due to factors such as patient age, the degree of root development, and infection severity. Protocol standardization and long-term follow-up are essential to enhance treatment predictability and success [46,47] (Figure 3).

### 2.2. Postnatal Stem Cell Therapy

Postnatal stem cell therapy is an evolving clinical approach in RETs, offering an alternative to conventional procedures like apexogenesis and apexification. The goal is to regenerate the pulp–dentin complex in necrotic teeth, especially those with immature roots and open apices [31].

The practical application involves isolating adult stem cells—commonly from dental pulp, apical papilla, or periodontal ligament—and delivering them into a disinfected root canal space. These stem cells are typically introduced along with a scaffold, such as a collagen matrix or a blood clot, to support cell attachment and differentiation [48].

Once placed, these cells can differentiate into odontoblast-like cells, fibroblasts, and endothelial cells, promoting dentin formation and revascularization of the pulp space. This contributes not only to continued root development but also to the restoration of pulp vitality, including sensory functions like temperature and pressure detection [49,50].

Among various sources, dental pulp stem cells (DPSCs) are especially promising due to their ease of isolation and capacity to promote dentinogenesis. Clinical protocols often combine these cells with bioactive molecules and controlled scaffolds to enhance tissue regeneration outcomes in immature teeth [48,49]. However, challenges remain in clinical translation, including maintaining cell viability during delivery, controlling differentiation, and preventing off-target migration. Success depends on the coordination of three key elements: viable stem cells, an appropriate scaffold, and inductive growth factors. When optimized, this triad can lead to effective regeneration and long-term tooth preservation [51,52].

### 2.3. Replacement Pulp Implantation

Replacement pulp implantation is a clinically focused RET strategy that involves inserting stem cell-loaded scaffolds into the disinfected root canal to regenerate pulp tissue. The scaffold acts as a structural support mimicking the extracellular matrix, enabling cell attachment, survival, and differentiation [53,54].

Biodegradable scaffolds—often made from collagen, fibrin, or synthetic polymers—are combined with growth factors such as VEGF or NGF to promote angiogenesis and neurogenesis. These elements are essential for re-establishing the pulp’s vascular and neural components. DPSCs or mesenchymal stem cells (MSCs) are seeded onto the scaffold before implantation [55,56].

Clinically, cells and scaffolds are delivered into the canal using precision-guided microsyringes or hydrogel carriers to ensure accurate placement and minimize procedural trauma. Hydrogels also enhance cell viability and retention within the canal space during implantation [35,36].

Autologous stem cells, sourced directly from the patient, are preferred to reduce immune reactions. However, when using allogenic cells, strict aseptic protocols are followed to prevent immune rejection or infection [57]. This method not only supports dentin and vascular tissue formation but also aims to fully restore pulp vitality, offering a regenerative alternative to traditional root canal therapy [53,58].

### 2.4. Scaffold Implementation

Scaffold implantation is a key component of RETs, providing a structural framework to support cell growth and tissue regeneration in necrotic teeth. In clinical practice, scaffolds are introduced into the disinfected root canal to mimic the extracellular matrix (ECM), facilitating angiogenesis and neurogenesis, both of which are essential for restoring pulp vitality [53].

Natural scaffolds (e.g., collagen and fibrin) offer a bioactive environment that supports cell attachment and signaling. Synthetic scaffolds, such as polylactic acid (PLA) and polyglycolic acid (PGA), provide customizable mechanical properties and controlled degradation rates, making them suitable for various clinical needs [59,60]. In practice, synthetic scaffolds may undergo surface modifications—such as coating with bioactive molecules—to enhance cell adhesion and biocompatibility, promoting more efficient tissue regeneration [55,61,62].

Matching scaffold degradation with tissue formation is critical. A scaffold that degrades too quickly or too slowly may trigger inflammation or disrupt tissue development [43,63]. Clinicians are increasingly incorporating nanoparticles (e.g., AgNPs, AuNPs, and BGNs) into scaffolds to enhance their function. For example, silver nanoparticles provide antimicrobial effects, while bioactive glass supports mineralization and dentin formation [55]. The selection of scaffold type is guided by clinical factors such as the tissue condition, required degradation profile, and immune response. When appropriately selected and delivered, scaffold implantation offers a reliable platform for promoting pulp regeneration and improving long-term treatment outcomes in regenerative endodontics [53].

### 2.5. Injectable Scaffold Delivery

Injectable scaffold delivery in RETs offers a minimally invasive and effective method to promote rapid pulp regeneration, especially for necrotic teeth. By eliminating the need for extensive surgical intervention, this technique reduces tissue disruption while enhancing healing outcomes [53,64,65].

In practice, injectable hydrogels—such as chitosan, collagen, or fibrin-based formulations—are delivered into the disinfected root canal using a syringe. These hydrogels are capable of adapting to the complex anatomy of the canal, forming a three-dimensional matrix that supports essential cellular activities. The scaffold facilitates cell migration, attachment, and proliferation, all of which are critical for the differentiation of DPSCs or mesenchymal stem cells (MSCs) into functional pulp tissue [66,67,68].

These scaffolds support key biological processes, helping integrate the newly formed pulp-like tissue with existing dentin and vascular structures. To enhance regenerative potential, bioactive molecules such as bone morphogenetic proteins (BMPs) and vascular endothelial growth factor (VEGF) are frequently incorporated into the hydrogel. These factors stimulate angiogenesis and odontoblast-like cell formation, both vital for the restoration of pulp vitality and long-term treatment success [69,70].

As these hydrogels degrade, they release embedded therapeutic molecules in a controlled manner, which sustains the regenerative environment and promotes revascularization and reinnervation. This contributes to the full restoration of the pulp–dentin complex, including the formation of nerves and blood vessels, which are key components for sensory function and immune defense [22,71,72,73].

**Figure 4:**
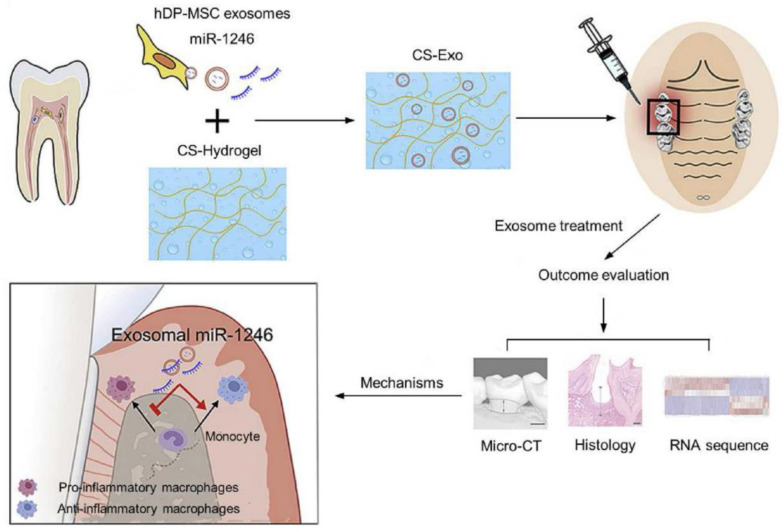
Injectable scaffolds show significant potential for minimally invasive pulp regeneration, promoting faster recovery and reducing complications. The scaffold is injected into the treatment site, where it promotes tissue regeneration by modulating the immune response, balancing pro- and anti-inflammatory macrophages. Evaluation methods such as micro-CT, histology, and RNA sequencing are used to assess the outcome of the treatment. Adopted from [58].

Recent advancements include the use of injectable chitosan hydrogels combined with stem cell-derived exosomes, which help modulate the immune response and further enhance the healing microenvironment. The clinical performance and regenerative progress of these injectable systems are typically monitored using advanced tools such as micro-CT imaging, histological analysis, and RNA sequencing, ensuring precise evaluation of scaffold behavior and tissue regeneration (Figure 4) [58].

### 2.6. Three-Dimensional Cell Printing

Three-dimensional (3D) cell printing represents a major advancement in RETs, offering highly accurate and patient-specific approaches for pulp–dentin regeneration [74]. This technique employs biocompatible materials—such as hydroxyapatite, bioglass, GelMA, and mineral trioxide aggregate (MTA)—which support cell proliferation and differentiation during tissue repair. Technologies like fused deposition modeling (FDM) and stereolithography (SLA) are widely used to fabricate scaffolds that closely replicate the anatomical architecture of the tooth, supporting seamless integration and healing [75,76,77].

In clinical and preclinical settings, 3D bioprinting allows for the creation of scaffolds tailored to a patient’s unique root canal morphology. These scaffolds enhance vascularization, neurogenesis, and overall tissue integration, offering distinct advantages in cases where traditional RETs may fall short. For instance, calcium silicate-based scaffolds combined with GelMA have demonstrated improved cell adhesion and odontogenic differentiation, which are key processes for regenerating the pulp–dentin complex in necrotic teeth. When stem cells and growth factors are embedded within these scaffolds, they further stimulate the formation of functional pulp and periapical tissues [39,75,76,77].

Table 3 outlines major 3D printing techniques applied in endodontics, detailing their materials, roles in tissue regeneration, and specific clinical applications. Hard scaffolds produced via FDM are suitable for structural support, while hydrogel-based printing is ideal for soft-tissue engineering, each addressing specific needs in pulp regeneration [42,78].

The ability to precisely regenerate lost pulp, including its vascular and neural components, makes 3D bioprinting a powerful option for managing necrotic teeth. This technique not only restores function and vitality but also improves aesthetic outcomes with minimally invasive procedures [41,42].

The integration of 3D bioprinting with computer-aided design (CAD) and manufacturing (CAM), using data from cone-beam computed tomography (CBCT), enables anatomically accurate scaffold fabrication. This combination enhances procedural planning, guided access, and treatment predictability. Additionally, it supports pre-surgical visualization and training, emphasizing the transformative impact of 3D printing technologies in modern endodontic practice [79,80,81].

**Table 3:** Comparison of 3D printing techniques in endodontics.

Type of 3D Printer	Materials Used	Role in Endodontics	Ref
SLA	Photosensitive resin	Precise layer-by-layer curing; ideal for creating surgical guides for guided endodontic access and pre-surgical planning (3D)	[14,82]
FDM	Thermoplastic filaments (PLA and ABS)	Cost effective for educational models and simulations in endodontic training (3D)	[14,41]
PolyJet/MultiJet Printing (MJP)	Photopolymer	Accurate, high-resolution models for surgical planning, such as in autotransplantation or root-end surgery (3D)	[83]
Digital Light Processing (DLP)	Photosensitive resin	Used for creating detailed anatomical models for guided surgical procedures (3D)	[84]
Selective Laser Sintering (SLS)	Powdered materials (e.g., metal and polymer)	Produces durable and complex structures; useful for creating surgical guides and models involving metallic components (3D)	[79]
ColorJet Printing (CJP)	Powder-based materials with binder	Primarily used for educational models and visualization aids in endodontic training, such as replicating anatomical features (3D)	[79]

### 2.7. Gene Therapy

Gene therapy in endodontics represents a groundbreaking approach to regenerating dental tissues through advanced genetic modifications, such as CRISPR/Cas9, which allows for precise gene editing to restore pulp vitality in teeth affected by disease or trauma [53]. By activating the genes responsible for dentin and vascular formation, gene therapy promotes the long-term survival of the pulp, aiming to regenerate a biological pulp–dentin complex rather than relying on inert materials to fill the canal [85,86,87]. It targets genes involved in cell growth, differentiation, and immune response, harnessing the body’s natural healing mechanisms, making it especially beneficial in cases of necrotic pulp with limited regenerative capacity [43,88].

Gene therapy encompasses both in vivo and ex vivo techniques. In vivo applications involve directly introducing genes, such as bone morphogenetic proteins (BMPs), into the damaged pulp, stimulating natural regenerative pathways for dentin and vessel formation without the need for invasive procedures. Ex vivo methods, on the other hand, modify stem cells outside the body, causing them to overexpress BMPs before reintroducing them into the root canal, where they act as bioactive scaffolds to enhance tissue regeneration [53]. These methods support the structural and functional restoration of the pulp-dentin complex, including sensory and immune functions, which are crucial for the vitality of the treated tooth.

**Figure 5:**
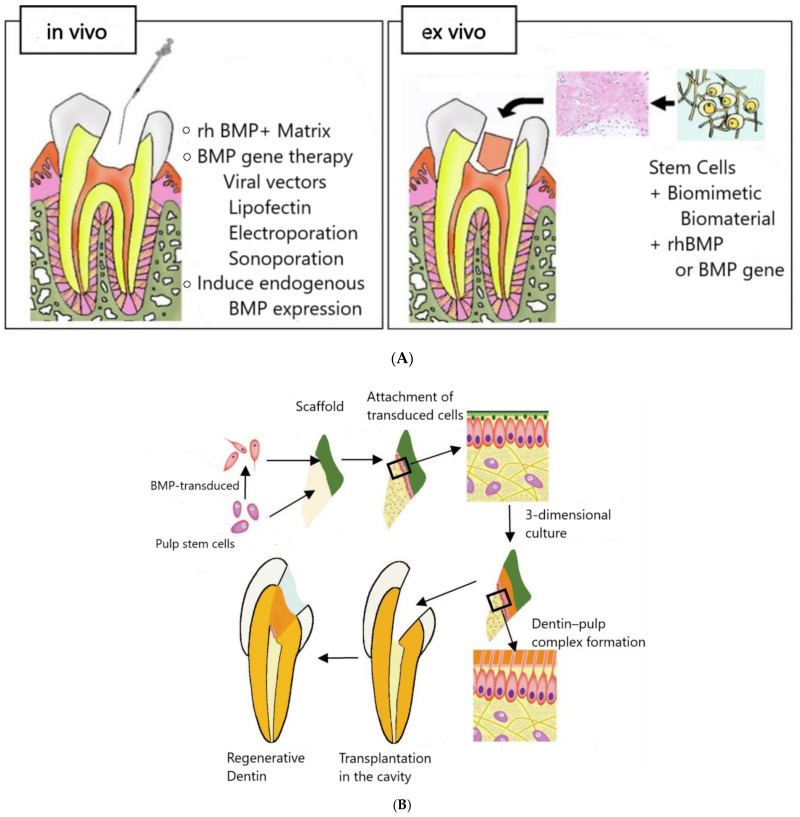

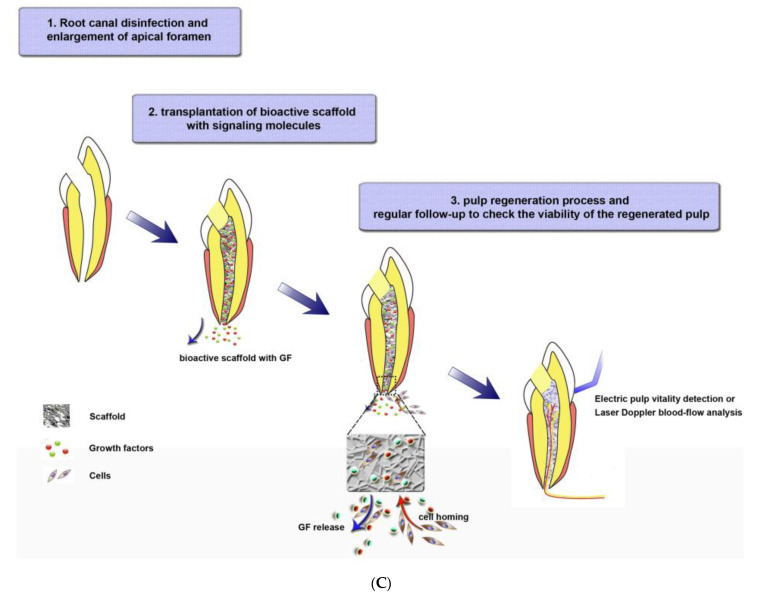
(**A**) Dentin regeneration techniques: in vivo methods use rhBMP, gene therapy, and electroporation to induce BMP expression; ex vivo approaches involve transplanting odontoblasts or stem cells with biomaterials and BMP genes into dentin matrices. (**B**) Regeneration process illustrating the steps from BMP transduction of pulp stem cells, their attachment to scaffolds, and the formation of a dentin–pulp complex in a 3-dimensional culture, ultimately leading to the regeneration and transplantation of dentin in dental cavities. (**C**) RE via cell homing strategy: (1) The process begins with disinfecting the root canal and enlarging the apical foramen to prepare for regeneration. (2) A bioactive scaffold is then implanted into the cleaned canal, releasing growth factors that attract cells critical for tissue formation. (3) As these cells migrate, proliferate, and differentiate within the scaffold, they contribute to the development of vital dental structures such as blood vessels, nerves, and dentin. Regular follow-ups are essential to monitor the health and integration of the regenerated pulp. Adopted from [43,47,53].

In RETs, integrating BMPs and other growth factors within 3D scaffolds enables controlled pulp regeneration by mimicking the natural pulp dentin structure. These scaffolds, which release therapeutic genes, create an environment conducive to the development of vital structures such as blood vessels and nerves. Figure 5 illustrates the techniques for in vivo BMP application, ex vivo cell transduction, and cell homing, all contributing to the creation of a bioactive environment that supports pulp regeneration.

Despite its potential, gene therapy faces several challenges, including the complexity of delivery methods, the precision of gene editing, off-target effects, and safety concerns. Achieving effective revascularization, essential for tissue survival, is particularly challenging due to the intricate anatomy of root canals, especially in the apical regions. Scaffold integration, crucial for supporting tissue regeneration, is also complicated by the complex canal structures, and infection control is critical, since engineered tissues are susceptible to infection, which could impair long-term success. These challenges underscore the need for continued research to refine delivery systems, optimize scaffold designs, and improve clinical efficacy in RETs [43,47,53,89].

While gene therapy holds immense promise for revolutionizing pulp regeneration and dentin repair in endodontics, it necessitates rigorous clinical validation and ethical considerations to ensure a balance between the potential benefits and the risks, guaranteeing safe and effective outcomes for patients’ dental health [90].

## 3. Stem Cells in RETs: Properties, Sources, and Types

In RETs, stem cells are pivotal for dental pulp regeneration due to their unique properties. Their plasticity enables them to differentiate into odontoblast-like cells, which are essential for forming a functional pulp–dentin complex in cases of necrotic pulp [91,92]. Stem cells, sourced from tissues like dental pulp or bone marrow, are expanded and differentiated under controlled conditions in bioreactors and then incorporated into biodegradable scaffolds. These scaffolds provide structural support within the damaged pulp, facilitating tissue regeneration.

Beyond simple tissue replacement, the post-implantation environment is optimized with the addition of growth factors and anti-inflammatory agents that enhance cell survival, promote innervation, and stimulate vascularization [93,94]. Recent advancements in genetic engineering, including CRISPR, offer the potential to tailor stem cell therapies to individual patients, aligning treatment strategies with the body’s natural healing processes and setting a new standard for sustainable outcomes in endodontics [95,96].

Moreover, the self-renewal ability of stem cells ensures a continuous supply of undifferentiated cells for pulp regeneration. DPSCs demonstrate robust self-renewal, regulated by key factors like TGF-β, BMPs, and FGFs, which are crucial for tissue repair and bioengineered pulp therapies [31,97]. Additionally, the multipotency of DPSCs allows them to differentiate into various cell types—odontoblasts, fibroblasts, and endothelial cells—all essential for rebuilding the pulp–dentin complex and restoring vital functions, such as sensory perception and immune defense [91,92,98]. This unique combination of regenerative capabilities positions stem cells as a cornerstone in the future of endodontic treatments.

Choosing the right source of cells is critical for the successful regeneration of the pulp–dentin complex. Autologous cells, harvested from a patient’s own tissues such as dental pulp or bone marrow, are highly preferred due to their reduced risk of immune rejection and superior integration with existing tissues. These cells are ideal for promoting the restoration of full pulp functionality and ensuring a more predictable regenerative outcome [99,100].

Allogenic cells, sourced from donors, offer a more readily accessible option but pose risks of immune rejection and raise ethical concerns related to tissue sourcing and compatibility. While allogenic cells may be beneficial in certain scenarios, their use requires careful consideration of immunological factors and potential complications.

Xenogeneic cells, derived from animals, are less commonly used but can offer unique insights in experimental and preclinical models. However, their application is fraught with challenges, including high immune rejection rates and ethical considerations, making them a less favorable choice for clinical use [101,102].

Table 4 provides a comprehensive comparison of autologous, allogenic, and xenogeneic cells, detailing their respective benefits, challenges, sources, and potential applications in the regeneration of the pulp–dentin complex. This overview serves to guide decision making in selecting the most suitable cell source for regenerative endodontic procedures.

In RETs, various types of stem cells provide unique advantages, these are summarized as follows:

### 3.1. Fetal Stem Cells

Fetal stem cells, including embryonic and umbilical cord stem cells, are highly pluripotent, meaning they can differentiate into a wide range of cell types. However, their clinical use is restricted due to ethical concerns and regulatory challenges [50,107,108]. Embryonic stem cells, with unlimited division potential, could theoretically regenerate entire dental pulp or dentin structures, but their application remains largely theoretical because of these ethical restrictions [34,103,109,110,111]. Blastocyst stem cells, derived from the early stages of embryonic development, also display high pluripotency and show promise in regenerating complex dental structures like pulp and dentin [112,113]. Although they offer vast potential, their use in RETs remains largely experimental due to ethical and technical considerations. Fetal stem cells, although more limited in versatility compared to their embryonic counterparts, are highly targeted in their differentiation, often maturing into odontoblast-like cells necessary for dentin regeneration. This specificity makes them particularly valuable in RETs aimed at dentin repair and pulp regeneration [114,115].

### 3.2. Umbilical Stem Cells

Umbilical cord stem cells, often used in blood disorder treatments, are increasingly studied for their potential in dental pulp regeneration. These cells offer an easier collection process and pose fewer ethical concerns than embryonic stem cells, making them a promising source for RETs [116,117,118].

Adult stem cells, particularly DPSCs, located in bone marrow and dental pulp, are crucial for RETs. DPSCs can differentiate into odontoblasts-like cells, facilitating pulp-dentin complex regeneration and moving beyond traditional root canal treatments toward regenerative solutions [119,120].

### 3.3. Adult Stem Cells

Adult stem cells, particularly DPSCs, are among the most critical sources for RETs. Located in dental pulp and bone marrow, DPSCs can differentiate into odontoblasts, enabling them to regenerate the pulp–dentin complex. These cells move beyond conventional root canal treatments, offering regenerative solutions that restore natural tooth vitality. In addition to DPSCs, other adult stem cells from sources like the periodontal ligament (PDLSCs) and the apical papilla (SCAP) also play important roles in RETs, contributing to the formation of vascularized and innervated pulp–dentin complexes [121].

### 3.4. Mesenchymal Stem Cells (MSCs)

A key feature of RETs is the interaction between MSCs and epithelial cells. MSCs, which can be derived from DPSCs, PDLSCs, and SCAP, are essential in forming the vascular and neural networks necessary for fully functional pulp [122,123]. These interactions, regulated by signaling pathways such as BMP, TGF-β, and Wnt, promote the differentiation of MSCs into odontoblast-like cells that form dentin [124,125]. Co-culturing MSCs with epithelial cells in biomimetic scaffolds further enhances regenerative outcomes by mimicking natural cellular interactions, thereby improving tissue regeneration and supporting future clinical applications in endodontics [126,127,128].

By leveraging the regenerative capabilities of these diverse stem cells, RETs aim to restore dental structures to their natural, functional state, moving beyond traditional approaches to root canal therapy and offering patients long-term, biologically based solutions for tooth repair.

## 4. Scaffolds in RETs

In RETs, scaffolds serve as essential three-dimensional frameworks that support cellular growth, differentiation, and tissue regeneration. These structures mimic the ECM, providing a supportive environment for the repair and restoration of the pulp–dentin complex [129]. Scaffolds are pivotal in enabling RETs by replacing diseased or necrotic pulp tissue with healthy, functional tissue. They promote revascularization and facilitate the repopulation of the root canal with stem cells and growth factors, thereby contributing to the restoration of tooth vitality and function [130,131].

More than passive frameworks, scaffolds play an active role in guiding tissue regeneration. They create a microenvironment that promotes cell adhesion, proliferation, and differentiation, which are vital processes for the formation of a fully functional pulp–dentin complex [132]. In addition, scaffolds support the integration of vascular and neural networks within regenerated tissues, which is critical for the longevity and sensory functionality of the treated teeth.

Advanced materials and technologies have significantly enhanced the regenerative potential of scaffolds [133,134,135] [Table 5]. Innovations range from natural scaffolds like collagen to bioceramic and hybrid designs, transforming endodontic practices into more biologically driven and sustainable approaches.

Among these innovations, 3D bioprinting has emerged as a cutting-edge technology that allows for the precise fabrication of scaffolds tailored to the complex anatomy of individual root canal systems. By integrating advanced imaging techniques such as CBCT, 3D-printed scaffolds can be customized for each patient, improving the scaffold fit, biological performance, and overall success of regeneration [136].

### 4.1. Natural Scaffolds

Naturally derived scaffolds such as collagen, chitosan, and alginate provide excellent biocompatibility and closely resemble the native ECM, supporting effective cell attachment, proliferation, and differentiation. These materials are particularly beneficial in patients with necrotic teeth, enhancing long-term pulp vitality and regeneration [137,138]. However, they are often characterized by rapid degradation and weak mechanical properties, requiring reinforcement for long-term use [138].

#### 4.1.1. Collagen

Known for its ability to recruit DPSCs, collagen facilitates the formation of pulp–dentin-like tissue. It has demonstrated success in clinical applications due to its close replication of the body’s ECM and ability to enhance cell attachment and proliferation [137]. However, it often requires chemical modification to improve its mechanical strength and structural durability.

#### 4.1.2. Chitosan

Valued for its inherent antibacterial properties, chitosan supports fibroblast and odontoblast activities, aiding pulp regeneration [37,139]. Despite its benefits, challenges such as inconsistent mechanical properties and pore size control limit its widespread application.

#### 4.1.3. Alginate

Alginate has demonstrated clinical success due to its biocompatibility and similarity to the ECM, supporting tissue integration and regeneration [137]. While it allows gentle gelation and supports cell encapsulation, it is prone to rapid degradation and offers limited mechanical strength, which may restrict its long-term performance unless reinforced.

### 4.2. Synthetic Scaffolds

Synthetic scaffolds, including those made from polylactic acid (PLA), polyglycolic acid (PGA), poly (lactic-co-glycolic acid) (PLGA), and various hydrogels, offer precise control over mechanical properties and degradation rates [38,55]. These materials are engineered to degrade in harmony with the pace of new tissue formation, ensuring continuous structural support throughout the regeneration process.

Their high customizability makes them adaptable to different stages of tissue healing and specific clinical requirements, such as damage severity, regeneration timelines, and the surrounding biological environment. However, they may require complex fabrication methods and surface modifications to enhance biocompatibility and minimize inflammatory responses. For instance, the application of functional coatings or specific chemical groups can improve cellular interactions and reduce immune reactions [140].

**Figure 6:**
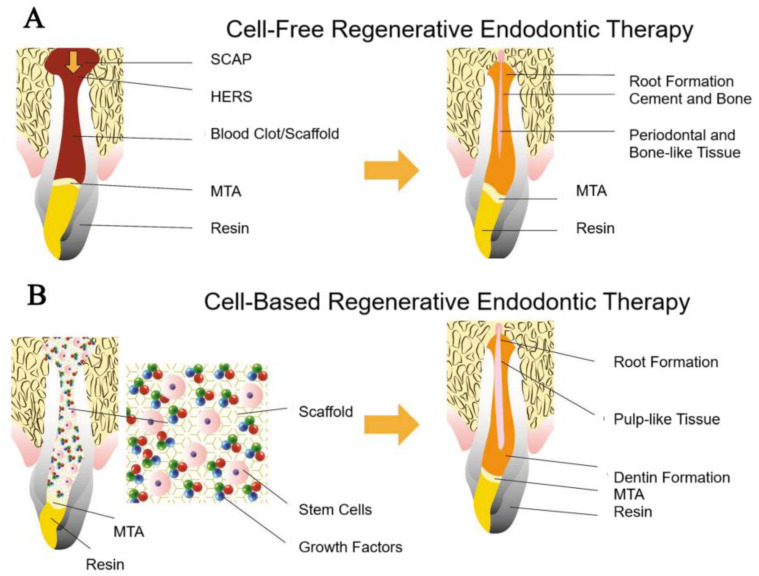
Schematic diagram illustrating the two Regenerative Endodontic Therapy approaches with the use of biomaterial scaffolds. (**A**) Cell-free Regenerative Endodontic Therapy: a blood clot or scaffold is introduced into the canal space, leading to the regeneration of periodontal and bone-like tissue. (**B**) Cell-based Regenerative Endodontic Therapy: stem cells, scaffolds, and growth factors are placed in the canal space, resulting in the regeneration of pulp-like tissue. The key difference is that the cell-based approach promotes true pulp regeneration, whereas the cell-free approach encourages repair tissue formation. SCAP: stem cells from the apical papilla; HERS: Hertwig’s epithelial root sheath; MTA: mineral trioxide aggregate. Adapted with permission [69].

Regardless of the scaffold type, all support regenerative processes by facilitating cell recruitment, differentiation, and tissue formation, which are key factors for restoring pulp vitality and function [69] (Figure 6).

Emerging approaches, such as hybrid scaffolds that combine synthetic and natural materials, are further enhancing outcomes. These hybrid systems merge the mechanical strength of synthetic polymers with the bioactivity of natural components, positioning them as promising tools for personalized Regenerative Endodontic Therapies.

### 4.3. Bioceramic Scaffolds

Scaffolds made from calcium phosphate, bioactive glass, and other bioceramics promote regeneration by mimicking the mineralized components of dental tissue. These materials enhance osteoconductivity and encourage DPSCs to differentiate into odontoblast-like cells. Bioceramic scaffolds provide robust structural integrity while releasing bioactive ions, such as calcium and phosphate, to stimulate tissue growth and repair.

Their ability to integrate with natural dental structures makes them particularly effective for restoring pulp vitality in necrotic teeth [141,142].

### 4.4. Hybrid Scaffolds and Advanced Techniques

Recent innovations include hybrid scaffolds that combine the biological properties of natural materials with the mechanical strength of synthetic ones. These scaffolds optimize the balance between biocompatibility and structural support, further enhancing their regenerative potential.

**Table 5:** Biomaterials used in endodontic tissue engineering: sources, properties, and applications.

Biomaterial	Source	Key Biochemical Components	Favorable Properties	Limitations	Specific Endodontic Applications	Mechanism/ Function	Target Regeneration	Ref
Host-derived biomaterial scaffolds								
Blood clot	Host derived	Fibrin	- Low cost - Clinical simplicity - Host compatibility	Instability, difficulties in invoking bleeding, and hemostasis	Scaffold for RE	Forms fibrin clot and supports cell migration	Pulp tissue regeneration	[3,47,143]
Autologous platelet concentrates	Autologous blood	TGF and VEGF	- Controlled release of growth factors - Host compatibility	Expensive, requires special equipment and reagents	Scaffold for RE	Promotes cell differentiation and tissue healing	Pulp and dentin regeneration	[47,143,144,145]
PRP	Autologous blood	Platelets and fibrin	- Controlled release of growth factors - Host compatibility	Comparatively expensive	Scaffold for pulp regeneration	Promotes stem cell homing and tissue repair	Pulp regeneration	[47,97,144,145,146]
PRF	Autologous blood	Platelets and fibrin	- Host compatibility	Time consuming, with special equipment required	Scaffold for pulp and dentin regeneration	Slow release of growth factors; supports tissue repair	Pulp and dentin regeneration	[97,146,147]
Decellularized ECM	Human/ animal tissues	ECM proteins And growth factors	- Host compatibility - Conducive to tissue growth	Time consuming, with difficult preparation	Scaffold for pulp-dentin regeneration	Mimics native ECM and promotes stem cell adhesion	Pulp and dentin regeneration	[147,148,149]
Nature-derived biomaterial scaffolds								
Collagen	Bovine/ porcine dermis or tendons	Type I and III collagen proteins	Biocompatible, biodegradable, and viscoelastic	Rapid degradation, weak mechanical strength, and shrinkage	Scaffold for pulp-dentin complex regeneration	Mimics natural ECM and supports cell adhesion and differentiation	Pulp and dentin regeneration	[47,143,150,151]
Chitosan	Derived from chitin (shrimp/crab shells)	N-acetyl glucosamine and glucosamine units	Host compatibility; biodegradable; antibacterial properties	Weak mechanical strength and shrinkage	Root canal disinfectant, pulp capping, and scaffold	Forms hydrogels, enhances tissue regeneration, and inhibits bacterial growth	Pulp capping and root regeneration	[47,143,152,153]
Alginate	Brown seaweed (Laminaria, Ascophyllum, and Macrocystis)	Sodium alginate (mannuronic and guluronic acids)	Host compatibility; inexpensive and supports nutrient exchange	Weak mechanical strength before cross-linking; shrinkage	Cell delivery, scaffold, and drug delivery system	Forms hydrogels that encapsulate stem cells or growth factors; supports controlled cell release	Pulp regeneration; scaffold for growth factors	[47,143,154,155]
Fibrin	Human blood (plasma)	Fibrinogen and thrombin	Host compatibility; creates fibrin clot	Requires clot formation; short-term support	Scaffold for RE	Creating a fibrin clot for cell adhesion and migration	Pulp tissue healing and vascularization	[156,157]
Hyaluronic acid	Animal connective tissue (skin and joints)	Hyaluronan polysaccharides	Biocompatible, retains moisture, and promotes tissue healing	Rapid degradation and weak mechanical strength	Pulp tissue hydration, ECM mimic, and scaffold	Retains moisture and promotes cell proliferation and migration	Pulp healing promotes angiogenesis	[158,159]
Silk fibroin	Silkworm cocoons (Bombyx mori)	Fibroin and sericin proteins	Biocompatible and promotes cell differentiation; strong scaffold	Limited availability and complicated processing	Scaffold for pulp and periodontal regeneration	Provides mechanical support and promotes differentiation of stem cells	Pulp and periodontal regeneration	[156,157]
Gelatin	Hydrolyzed collagen from animal skin/bone	Denatured collagen (Type I)	Biodegradable, forms hydrogels, and enhances cell proliferation	Rapid degradation and weak mechanical strength	Scaffold for cell culture; TE	Forms hydrogels and enhances cell attachment and proliferation	Cell proliferation, scaffold for pulp TE	[160,161,162]
Dextran	Produced by bacterial fermentation (Leucistic)	Glucose polymer (α-1,6 glycosidic linkages)	Biocompatible, slowly degrades, and supports prolonged healing	Limited in regenerative capabilities for some tissues	Drug delivery; scaffold for growth factors	Biocompatible; used as a carrier for growth factors or drugs	Controlled drug delivery; tissue repair	[163,164]
Synthetic biomaterial scaffolds								
Hydraulic calcium	Synthetic	Tricalcium silicate-based materials	Biocompatible	Tooth discoloration	Scaffold for pulp regeneration	Promotes odontogenic cell differentiation and supports mineralization	Pulp regeneration	[165,166,167]
Synthetic polymers	Synthetic	- PLA - PGA	Biodegradable, precisely modifiable physicochemical properties	Relatively slow degradation rate and potential host response	Scaffold for pulp regeneration	Provides mechanical support and customizes pore size for stem cell colonization	Pulp regeneration	[168,169]
Synthetic hydrogel	Synthetic	- Gelatin meth acryloyl - Peptide based	Biocompatible, injectable, and supports self-assembly	Slow gelation; UV light required for some hydrogels may cause cell death	Scaffold for pulp regeneration	Promotes cell proliferation and mimics ECM	Pulp regeneration	[170,171,172]

## 5. Growth Factors in RETs

Growth factors are critical in regulating cellular activities such as proliferation, differentiation, migration, and tissue repair, positioning them as key elements in regenerating the pulp–dentin complex [88]. These biologically active molecules exert their effects by activating specific signaling pathways, interacting with ECM components, and directing the behavior of stem cells within engineered tissues.

**Figure 7:**
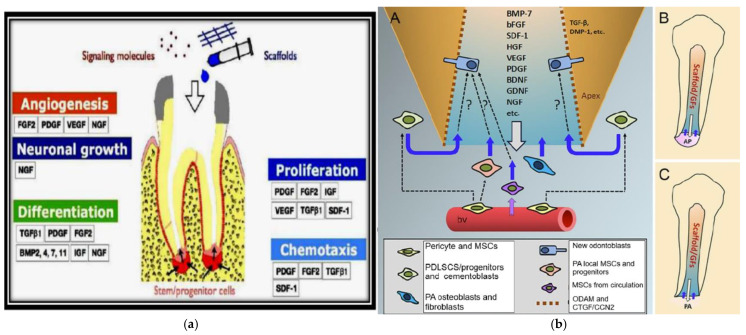
(**a**) Depicts the interaction of growth factors with stem cells and pericytes in dental pulp regeneration, emphasizing angiogenesis and tissue formation (**b**) Showcases a detailed schematic of regenerative endodontic therapy, with a focus on growth factor deployment, stem cell interaction, and scaffold implantation to foster dental pulp regeneration [174,175].

In endodontics, growth factors have been extensively studied for their ability to enhance tissue healing and regeneration (Table 6). By orchestrating cellular and molecular processes, they facilitate the formation of functional pulp–dentin structures and improve the outcomes of regenerative therapies [173,174,175] (Figure 7).

### 5.1. Signal Activation

Growth factors activate pathways such as BMP, TGF-β, and VEGF, which are critical for odontogenesis, angiogenesis, and tissue integration.

### 5.2. Stem Cell Modulation

They influence stem cell proliferation and differentiation, promoting the development of odontoblast-like cells necessary for dentin formation.

The strategic use of growth factors in regenerative endodontic procedures (REPs) can significantly accelerate the healing process, improve tissue functionality, and ensure long-term success in restoring tooth vitality.

## 6. Challenges and Future Directions in RETs

RETs face several challenges, particularly in regenerating nerve and vascular tissues within the dental pulp. The regeneration of nerve tissues is inherently complex due to the intricate microenvironment of the pulp and the limited regenerative capacity of neural elements. Functional integration of newly formed neural pathways requires precise differentiation of stem cells into neurons and alignment of new nerve fibers with existing ones, which remain significant barriers [4,61].

Future directions should prioritize the development of advanced biomaterials that emulate the native ECM of neural tissues. These biomaterials must provide both structural support and biofunctional cues to facilitate nerve regeneration. Techniques such as gene therapy and the incorporation of nerve growth factors (NGFs) can target specific signaling pathways to enhance neural differentiation and maturation. Additionally, micro- and nanotechnologies offer promising solutions, enabling the fabrication of scaffolds with topographical features that guide nerve growth, alignment, and functional integration [69,214].

Another critical challenge is the re-establishment of a robust blood supply, which is essential for the survival and functionality of regenerated pulp tissue. Insufficient vascularization within engineered tissues can lead to hypoxia, nutrient deficiencies, and compromised cell survival and differentiation [12]. Incorporating angiogenic factors, such as VEGF and angiopoietin, into scaffolds has shown potential in promoting blood vessel formation and maturation. Furthermore, bioprinting technologies can fabricate scaffolds with predesigned vascular channels, which, when seeded with endothelial cells, form functional vascular networks. MSCs also hold promise for enhancing vascularization due to their ability to differentiate into vascular cells and support endothelial growth [215,216].

Future research must focus on biomaterials that not only replicate the ECM structure but also actively encourage vascular growth and stabilization. Integrating bioprinting with angiogenic factors and further exploring MSC-based approaches could address current limitations, advancing the efficacy of vascularized pulp regeneration [69,214].

In addition to biological and technical challenges, commercialization and regulatory approval represent significant hurdles for the clinical translation of RETs. High costs associated with cell isolation, scaffold fabrication, and growth factor production have been identified as major barriers limiting the accessibility and widespread adoption of regenerative therapies [29,30]. Moreover, strict regulatory requirements for ensuring safety, efficacy, and quality control, along with the lack of standardized clinical protocols, further complicate the transition from laboratory research to clinical application, as emphasized in both reviews [29,30]. Addressing these commercial and regulatory challenges will require the development of cost-effective manufacturing processes, scalable production methods, and robust clinical trial designs. Collaboration between researchers, clinicians, industry partners, and regulatory agencies will be essential to facilitate the successful and practical translation of RETs into routine dental care.

## 7. Conclusions

Tissue engineering has revolutionized endodontic treatment, shifting the paradigm from preserving tooth structure to regenerating functional, vital dental tissues. This approach is especially impactful for immature teeth with necrotic pulps, where RETs can restore vitality, reduce fracture risks, and improve the longevity of treated teeth. Unlike traditional endodontic therapies focused on infection removal and root canal filling, regenerative strategies aim to heal and regenerate the pulp–dentin complex using stem cells, biocompatible scaffolds, and growth factors.

Innovations in root canal revascularization have accelerated with the application of adult stem cells, scaffold technologies, and morphogens like BMPs, aligning with deeper insights into molecular pathways underlying dental regeneration. These advancements enhance cellular differentiation and guide natural tissue regeneration processes, establishing a foundation for more effective and biologically aligned therapies.

A key area of recent focus in RETs is neuroregeneration, which targets the restoration of nerve fibers within engineered pulp tissues. Research highlights the role of neurotrophic factors and optimized scaffold designs in facilitating neural growth. These advancements aim to restore sensory functions and mimic the pain responses of natural teeth, improving the physiological outcomes of RE.

Additionally, the integration of gene therapy into RE represents an exciting frontier. Gene-based approaches, combined with scaffolds designed to support cellular growth and tissue repair, could significantly enhance the scope of RETs. However, translating these breakthroughs into routine clinical practice demands extensive research to ensure safety, predictability, and widespread applicability.

In summary, RETs leverages the inherent healing capacity of the dentin–pulp complex, integrating stem cells, molecular signals, and biomaterials to achieve true tissue restoration. Future success in RETs will depend on continued exploration of the cellular and molecular mechanisms driving regeneration and fostering collaboration between researchers and clinicians. These efforts will establish RETs as a mainstream therapeutic option, offering improved patient outcomes, reduced complications, and enhanced long-term oral health. As the field advances, tissue engineering’s transformative impact will solidify its role as a cornerstone of modern endodontic therapy, significantly improving the quality of life for patients.

## Figures and Tables

**Table 1:** Summary of RET clinical cases in adult patients.

Case Study	Patient Demographics	Tooth Condition	Procedure	Follow-Up and Outcomes	Ref
Case 1	11-year-old girl	Tooth #8, necrotic with closed apex	Apical revascularization with sodium hypochlorite, calcium hydroxide, MTA, and collagen membrane	18-month follow-up; asymptomatic, with resolution of periapical radiolucency	[17]
Case 2	14-year-old female	Tooth #9, necrotic with closed apex	Apical revascularization with sodium hypochlorite, calcium hydroxide, MTA, and collagen membrane	22-month follow-up; asymptomatic, with resolved periapical radiolucency	[17]
Case 3	23-year-old female	Teeth #7 and #8, necrotic with periapical lesions	Chemo-mechanical debridement, triple-antibiotic paste, and MTA over blood clot	12-month follow-up; symptom reduction, with decreased periapical radiolucency size	[18]
Case 4	21-year-old male	Necrotic mature teeth, symptomatic apical periodontitis	Sodium hypochlorite irrigation, calcium hydroxide, MTA on collagen membrane, and evoked bleeding	10-month follow-up; radiographic healing, asymptomatic	[19]
Case 5	9-year-old girl	Necrotic immature permanent central incisor with sinus tract	Regenerative treatment with Ca (OH)_2_, induced bleeding, and MTA over blood clot	2.5-year follow-up; continued root development, root wall thickening, and apical closure	[20]
Case 6	11-year-old boy	Immature dens invaginatus with periapical periodontitis	Pulp revascularization with NaOCl irrigation, triple-antibiotic paste, and glass ionomer cement	Complete healing, apex closure, and root wall thickening	[21]
Case 7	9-year-old boy	Immature mandibular molar with apical periodontitis	Revascularization with platelet-rich plasma and blood clot	Successful apical healing and tissue regeneration	[22]
Case 8	12-year-old boy	Immature necrotic tooth with periapical radiolucency	Revascularization with triple-antibiotic paste and MTA over blood clot	24-month follow-up; apex closure and root wall thickening	[23]
Case 9	8-year-old boy	Necrotic immature permanent tooth with apical abscess	Revascularization with triple-antibiotic paste, induced bleeding, and MTA seal	11-month follow-up; complete apexogenesis and healing	[24]
Case 10	15-year-old boy	Nonvital immature anterior tooth with periapical lesion	Revascularization with PRP and collagen sponge	12-month follow-up; apical closure and root elongation	[25]
Case 11	14-year-old female	Immature maxillary central incisors, necrotic	Regenerative endodontic treatment with triple-antibiotic paste, induced bleeding, and MTA seal	6-year follow-up; root functionality, healed apices, and discoloration issues	[26]
Case 12	12-year-old boy	Teeth #8 and #9, necrotic pulp, apical periodontitis	PRF-based regenerative endodontic procedure; triple-antibiotic paste, Bio dentine, glass ionomer, and PRF scaffold	30-month follow-up; arrested external root resorption (ERR), apical closure, and asymptomatic state	[27]
Case 13	12-year-old girl	Mandibular left second premolar with chronic abscess and incomplete root development	Sodium hypochlorite irrigation, tri-antibiotic paste, blood clot scaffold, MTA, and glass ionomer cement	18-month follow-up; resolution of periapical radiolucency, root maturation, and asymptomatic tooth	[28]
Case 14	9-year-old girl	Immature, traumatized maxillary central incisor with sinus tract	Minimal instrumentation, sodium hypochlorite irrigation, calcium hydroxide paste, induced bleeding, and MTA placement	2.5-year follow-up; progressive thickening of root walls, apical closure, asymptomatic condition, and sinus tract healing	[29]
Case 15	12-year-old boy	Tooth #8, post-trauma, symptomatic apical periodontitis with ERR	Triple-antibiotic paste, induced bleeding, PRF scaffold, glass ionomer, and Biodentine	24-month follow-up; significant healing, apical closure, arrested ERR, and asymptomatic condition	[30]

**Table 2:** Techniques for RETs.

Technique	Description	Advantages	Disadvantages	Ref
Root canal revascularization	Opening the tooth apex up to 1 mm to allow blood flow into the root canals	Low chance of immune system rejection; reduced risk of transmitting pathogens.	Few documented cases; risk of tissue death if reinfection occurs.	[32]
Stem cell therapy	Injecting autologous or allogenic stem cells or cell mixtures into the tooth via a matrix	Quick; Straightforward delivery with minimal discomfort; cells are easy to obtain.	Low survival rate for the cells; does not generate fully functional pulp; high complication risk.	[33,34]
Pulp implant	Cultivating pulp tissue in sheets and surgically implanting it	Cell sheets are relatively easy to grow in the lab; more stable than injecting individual cells.	Limited size due to lack of blood flow; requires precise adaptation to root canal shape.	[35,36]
Scaffold implant	Seeding pulp cells on a 3D scaffold for surgical implantation	Provides a framework for cell structure Some materials may support new blood vessel formation	Low cell viability post-implantation must fit accurately within the root canal	[37,38]
3D cell printing	Using inkjet-style devices to place cell layers in a hydrogel for surgical placement	Enables precise positioning of different types of cells	Needs exact fit for the root canal; effectiveness in living organisms remains unproven in early research	[39,40]
Injectable scaffolds	Delivery of hydrogels or cell-laden hydrogels via injection	Simple to apply; may act as a scaffold substitute to support tissue regeneration	Limited control over the tissue development process; low cell viability; effectiveness not yet validated in early trials	[41,42]
Gene therapy	Transferring mineralizing genes to vital pulp cells of necrotic or symptomatic teeth	Potentially eliminates the need for traditional root canal procedures and might reduce the need for stem cell transplants	Many cells in damaged teeth are nonviable; challenging to control; potential health risks; lacks FDA approval	[32,43]

**Table 4:** Comparison of cell types for pulp–dentin complex regeneration.

Type of Cells	Benefits/ Properties	Considerations/ Challenges	Sources	Potential Applications	Immune Response	Ref
Autologous cells (host’s own cells)	- Reduced immune response - Enhanced integration	- Limited availability in very ill or elderly patients - Requires harvesting from the patient, which can lead to donor site morbidity	- Dental pulp - Bone marrow - Adipose tissue	- Direct pulp capping - Full pulp regeneration	- Low risk of immune rejection - Autologous cells are tolerated by the host immune system due to being derived from the same individual, resulting in minimal inflammation and a low immune response	[31,103,104]
Allogenic cells (donor cells)	- Off-the-shelf availability - Cost efficient	- Potential immune reactions - Ethical and legal constraints on using human cell lines	- Umbilical cord blood - Donor bone marrow	- Pulp–dentin complex regeneration - Enhanced healing in mature teeth with apical lesions	- Moderate to high risk of immune rejection - Allogenic cells can induce immune responses due to foreign antigens, leading to inflammation; cytokines like TNF-α and IFN-γ are involved, which can compromise the regeneration process	[104,105,106]
Xenogenic cells (cells from different species)	- Avoids ethical issues related to human cells	- High potential for immune rejection - Ethical and immunological challenges - Risk of transmitting zoonotic diseases	- Animal tissues (e.g., pig tooth pulp)	- Experimental models for tissue regeneration - Potential future clinical applications if immune and ethical challenges are addressed	- High risk of immune rejection - Xenogeneic cells often provoke a strong immune response due to cross-species transplantation; these require significant immunosuppression to prevent rejection	[31,67,104]

**Table 6:** Essential growth factors and morphogens in dentin that facilitate regeneration and repair.

Growth Factor	Primary Source	Regenerative Function	Ref
TGF-β1	Dentin matrix-activated TH1 cells NK cells	Promotes the initial differentiation of odontoblasts and supports the formation of tertiary dentin.	[31,176,177,178,179,180]
TGF-β2	Platelets Macrophages Bone	Enhances the differentiation of DPSCs into cells capable of mineralizing dentin.	[31,176,177,181]
TGF-β3	Platelets Macrophages Bone	Stimulates the differentiation of odontoblasts, aiding in dentin formation.	[31,176,182,183]
BMP-2	Bone Cartilage	Stimulates odontoblast differentiation in both laboratory and animal models and enhances alkaline phosphatase activity and DSPP induction.	[176,184,185,186]
BMP-4	Bone Cartilage	Promotes odontoblast differentiation and dentin matrix formation.	[176,184,187]
BMP-7	Bone tissue Kidneys	Encourages the mineralization of DPSCs, enhancing their ability to form hard tissue.	[176,188,189,190]
IGF-1	Liver Local tissues	Promotes the growth and mineralizing differentiation of DPSCs and SCAP.	[176,191,192,193,194]
Hepatocyte Growth Factor	Liver Released during tissue injury	Facilitates the migration, proliferation, and survival of MSCs in the dental pulp.	[176,195,196]
VEGF	Cells in hypoxic conditions	Induces the formation of new blood vessels, promoting healing and tissue regeneration in dental tissues.	[176,178,197]
Adrenomedullin	Bone marrow Injured tissues	Supports odontoblastic differentiation through signaling pathways that activate p38.	[176,198,199,200]
FGF-2	Pituitary Adrenal glands	Promotes the migration and growth of stem cells, as well as the formation of blood vessels.	[176,197,201]
Platelet-Derived Growth Factor	Platelets Endothelial cells Placenta	Stimulates angiogenesis, enhances MSC migration, and modulates the process of odontoblastic differentiation.	[176,177,180,202,203,204]
Epidermal Growth Factor	Submaxillary glands Keratinocytes	Enhances the neurogenic differentiation of DPSCs and promotes healing of damaged tissues.	[31,176,197,205,206]
Placenta Growth Factor	Placenta	Facilitates the growth of blood vessels and supports the differentiation of MSCs into osteogenic cells.	[176,197,207,208]
Brain-Derived Neurotrophic Factor	Brain tissue Neurons	Promotes the survival and growth of neurons, encouraging their regeneration and axonal growth.	[192,209]
Glial Cell Line-Derived Neurotrophic Factor	Skeletal muscle Central nervous system	Stimulates nerve regeneration and supports the survival and proliferation of pulp cells during tissue repair.	[192,210,211,212]
Growth/Differentiation Factor 15	Nerve tissue Various cell types	Supports the regeneration and maintenance of neuronal cells, playing a key role in post-injury recovery.	[192,213]
NGF	Secreted by neurons Target tissue	Essential for the survival and regeneration of neurons, promoting recovery after nerve injury.	[31]
CSF	A wide range of cells	Stimulates the proliferation of specific stem cells, supporting tissue repair and regeneration.	[31]
EGF	Submaxillary glands	Promotes the proliferation of various cell types, including epithelial, glial, and mesenchymal cells, aiding wound healing.	[31]
FGF	A wide range of cells	Encourages the proliferation of a variety of cell types, supporting tissue repair and regeneration.	[31]
IGF	Liver Variety of cells	Promotes cell growth and differentiation across various tissues, supporting overall tissue regeneration.	[31]

## Data Availability

No new data were created or analyzed in this study.

## References

[1] Soares D.G., Bordini E.A., Swanson W.B., de Souza Costa C.A., Bottino M.C. (2021). Platform technologies for regenerative endodontics from multifunctional biomaterials to tooth-on-a-chip strategies. Clin. Oral Investig..

[2] Boyd R.C. (2019). Basic endodontic therapy. Wiggs’s Veterinary Dentistry: Principles and Practice.

[3] Howard D., Buttery L.D., Shakesheff K.M., Roberts S.J. (2008). Tissue engineering: Strategies, stem cells and scaffolds. J. Anat..

[4] Widbiller M., Schmalz G. (2021). Endodontic regeneration: Hard shell, soft core. Odontology.

[5] Al-Helou N., Fuks A.B., Moskovitz M., Tickotsky N. (2023). Contemporary Endodontics for Children and Adolescents.

[6] Cymerman J.J., Nosrat A. (2020). Regenerative endodontic treatment as a biologically based approach for non-surgical retreatment of immature teeth. J. Endod..

[7] Sheehy E.J., Kelly D.J., O’Brien F.J. (2019). Biomaterial-based endochondral bone regeneration: A shift from traditional tissue engineering paradigms to developmentally inspired strategies. Mater. Today Bio.

[8] Ahrendt G., Chickering D.E., Ranieri J.P. (1998). Angiogenic growth factors: A review for tissue engineering. Tissue Eng..

[9] Matichescu A., Ardelean L.C., Rusu L.C., Craciun D., Bratu E.A., Babucea M., Leretter M. (2020). Advanced biomaterials and techniques for oral tissue engineering and regeneration—A review. Materials.

[10] Doblado L.R., Martínez-Ramos C., Pradas M.M. (2021). Biomaterials for neural tissue engineering. Front. Nanotechnol..

[11] Khurshid Z., Alnaim A.J.A., Alhashim A.A.A., Imran E., Adanir N. (2022). Future of decellularized dental pulp matrix in regenerative endodontics. Eur. J. Dent..

[12] Sugiaman V.K., Jeffrey Naliani S., Pranata N., Djuanda R., Saputri R.I. (2023). Polymeric scaffolds used in dental pulp regeneration by tissue engineering approach. Polymers.

[13] Yang Q., Zheng W., Zhao Y., Shi Y., Wang Y., Sun H., Xu X. (2024). Advancing dentin remineralization: Exploring amorphous calcium phosphate and its stabilizers in biomimetic approaches. Dent. Mater..

[14] Huang T.H., Chen J.Y., Suo W.H., Shao W.R., Huang C.Y., Li M.T., Li Y.Y., Li Y.H., Liang E.L., Chen Y.H. (2024). Unlocking the Future of Periodontal Regeneration: An Interdisciplinary Approach to Tissue Engineering and Advanced Therapeutics. Biomedicines.

[15] Farjaminejad R., Farjaminejad S., Hasani M., Garcia-Godoy F., Sayahpour B., Marya A., Jamilian A. (2025). The Role of Tissue Engineering in Orthodontic and Orthognathic Treatment: A Narrative Review. Oral.

[16] El Gezawi M., Wölfle U.C., Haridy R., Fliefel R., Kaisarly D. (2019). Remineralization, regeneration, and repair of natural tooth structure: Influences on the future of restorative dentistry practice. ACS Biomater. Sci. Eng..

[17] Digka A., Sakka D., Lyroudia K. (2020). Histological assessment of human regenerative endodontic procedures (REP) of immature permanent teeth with necrotic pulp/apical periodontitis: A systematic review. Aust. Endod. J..

[18] Paryani K., Kim S.G. (2013). Regenerative endodontic treatment of permanent teeth after completion of root development: A report of 2 cases. J. Endod..

[19] Saoud T.M., Sigurdsson A., Rosenberg P.A., Lin L.M., Ricucci D. (2014). Treatment of a large cystlike inflam- matory periapical lesion associated with mature necrotic teeth using regenerative endodontic therapy. J. Endod..

[20] Murray P.E., Garcia-Godoy F., Hargreaves K.M. (2007). Regenerative endodontics: A review of current status and a call for action. J. Endod..

[21] Krupińska A.M., Skośkiewicz-Malinowska K., Staniowski T. (2021). Different approaches to the regeneration of dental tissues in regenerative endodontics. Appl. Sci..

[22] Yang J., Zhao Y., Qin M., Ge L. (2013). Pulp revascularization of immature dens invaginatus with periapical periodontitis. J. Endod..

[23] Jazayeri H.E., Lee S.M., Kuhn L., Fahimipour F., Tahriri M., Tayebi L. (2020). Polymeric scaffolds for dental pulp tissue engineering: A review. Dent. Mater..

[24] Liu H., Lu J., Jiang Q., Haapasalo M., Qian J., Tay F.R., Shen Y. (2022). Biomaterial scaffolds for clinical procedures in endodontic regeneration. Bioact. Mater..

[25] Kouhi M., de Souza Araújo I.J., Asa’ad F., Zeenat L., Bojedla S.S.R., Pati F., Zolfagharian A., Watts D.C., Bottino M.C., Bodaghi M. (2024). Recent advances in additive manufacturing of patient-specific devices for dental and maxillofacial rehabilitation. Dent. Mater..

[26] Shi X., Hu X., Jiang N., Mao J. (2025). Regenerative endodontic therapy: From laboratory bench to clinical practice. J. Adv. Res..

[27] Srisuwan T., Tilkorn D.J., Wilson J.L., Morrison W.A., Messer H.M., Thompson E.W., Abberton K.M. (2000). Molecular aspects of tissue engineering in the dental field. Periodontology.

[28] Ma Y., Xie L., Yang B., Tian W. (2019). Three-dimensional printing biotechnology for the regeneration of the tooth and tooth-supporting tissues. Biotechnol. Bioeng..

[29] Bansal R., Jain A., Mittal S. (2015). Current overview on challenges in regenerative endodontics. J. Conserv. Dent. Endod..

[30] Etezadkeyhan P. (2024). Recent Advances in Regenerative Endodontics: Clinical Applications and Challenges. J. Oral Dent. Health Nexus.

[31] Saoud T.M., Martin G., Chen Y.H., Chen K.L., Chen C.A., Songtrakul K., Malek M., Sigurdsson A., Lin L.M. (2016). Treatment of mature permanent teeth with necrotic pulps and apical periodontitis using regenerative endodontic procedures: A case series. J. Endod..

[32] Jung C., Kim S., Sun T., Cho Y.B., Song M. (2019). Pulp-dentin regeneration: Current approaches and challenges. J. Tissue Eng..

[33] Duncan H.F., Smith A.J., Fleming G.J.P., Cooper P.R. (2016). Epigenetic modulation of dental pulp stem cells: Implications for regenerative endodontics. Int. Endod. J..

[34] Park Y.J., Cha S., Park Y.S. (2016). Regenerative applications using tooth derived stem cells in other than tooth regeneration: A literature review. Stem Cells Int..

[35] Farjaminejad S., Farjaminejad R., Garcia-Godoy F. (2024). Nanoparticles in Bone Regeneration: A Narrative Review of Current Advances and Future Directions in Tissue Engineering. J. Funct. Biomater..

[36] Ercal P., Pekozer G.G. (2020). A current overview of scaffold-based bone regeneration strategies with dental stem cells. Adv. Exp. Med. Biol..

[37] Zhang F., King M.W. (2020). Biodegradable polymers as the pivotal player in the design of tissue engineering scaffolds. Adv. Healthc. Mater..

[38] Wang X., Krebbers J., Charalambous P., Machado V., Schober A., Bosse F., Müller H.W., Unsicker K. (2015). Growth/differentiation factor-15 and its role in peripheral nervous system lesion and regeneration. Cell Tissue Res..

[39] Liao W., Xu L., Wangrao K., Du Y., Xiong Q., Yao Y. (2019). Three-dimensional printing with biomaterials in craniofacial and dental tissue engineering. PeerJ.

[40] Farjaminejad R., Farjaminejad S., Nucci L., d’Apuzzo F., Grassia V., Majidi K., Jamilian A. (2024). 3D Printing Approach in Maxillofacial Surgery in Iran: An Evaluation Using the Non-Adoption, Abandonment, Scale-Up, Spread, and Sustainability (NASSS) Framework. Appl. Sci..

[41] Thalakiriyawa D.S., Dissanayaka W.L. (2024). Advances in regenerative dentistry approaches: An update. Int. Dent. J..

[42] Peters O.A., Paranjpe A., Gaudin A. (2021). Dentine–pulp complex regeneration. Regenerative Approaches in Dentistry: An Evidence-Based Perspective.

[43] Gathani K.M., Raghavendra S.S. (2016). Scaffolds in regenerative endodontics: A review. Dent. Res. J..

[44] Cotti E., Mereu M., Lusso D. (2008). Regenerative treatment of an immature, traumatized tooth with apical periodontitis: Report of a case. J. Endod..

[45] Lin L.M., Ricucci D., Huang G.J. (2014). Regeneration of the dentine–pulp complex with revitalization/revascularization therapy: Challenges and hopes. Int. Endod. J..

[46] Hargreaves K.M., Diogenes A., Teixeira F.B. (2013). Treatment options: Biological basis of regenerative endodontic procedures. Pediatr. Dent..

[47] Martin G., Ricucci D., Gibbs J.L., Lin L.M. (2013). Histological findings of revascularized/revitalized immature permanent molar with apical periodontitis using platelet-rich plasma. J. Endod..

[48] Dissanayaka W.L., Zhang C. (2020). Scaffold-based and scaffold-free strategies in dental pulp regeneration. J. Endod..

[49] Shah R., Shimpi M. (2020). Clinical Progresses in Regenerative Dentistry and Dental Tissue Engineering. Current Advances in Oral and Craniofacial Tissue Engineering.

[50] Capparè P., Tetè G., Sberna M.T., Panina-Bordignon P. (2020). The emerging role of stem cells in regenerative dentistry. Curr. Gene Ther..

[51] Soudi A., Yazdanian M., Ranjbar R., Tebyanian H., Yazdanian A., Tahmasebi E., Keshvad A., Seifalian A. (2021). Role and application of stem cells in dental regeneration: A comprehensive overview. Excli J..

[52] Xia H., Li X., Gao W., Fu X., Fang R.H., Zhang L., Zhang K. (2018). Tissue repair and regeneration with endogenous stem cells. Nat. Rev. Mater..

[53] Huang G.T.J. (2008). A paradigm shift in endodontic management of immature teeth: Conservation of stem cells for regeneration. J. Dent..

[54] Lee B.N., Moon J.W., Chang H.S., Hwang I.N., Oh W.M., Hwang Y.C. (2015). A review of the regenerative endodontic treatment procedure. Restor. Dent. Endod..

[55] Yang J., Yuan G., Chen Z. (2016). Pulp regeneration: Current approaches and future challenges. Front. Physiol..

[56] Alghutaimel H.A.H. (2021). Decellularised Dental Pulp Tissue as a Potential Biological Scaffold for Endodontic Tissue Regeneration. Ph.D. Thesis.

[57] Woloszyk A. (2016). The Angiogenic Potential of Human Dental Pulp Stem Cells Cultured on 3D Silk Scaffolds. Ph.D. Thesis.

[58] Lu H., Mu Q., Ku W., Zheng Y., Yi P., Lin L., Li P., Wang B., Wu J., Yu D. (2024). Functional extracellular vesicles from SHEDs combined with gelatin methacryloyl promote the odontogenic differentiation of DPSCs for pulp regeneration. J. Nanobiotechnol..

[59] Luzuriaga J., Polo Y., Pastor-Alonso O., Pardo-Rodríguez B., Larrañaga A., Unda F., Sarasua J.R., Pineda J.R., Ibarretxe G. (2021). Advances and perspectives in dental pulp stem cell based neuroregeneration therapies. Int. J. Mol. Sci..

[60] Yan H., De Deus G., Kristoffersen I.M., Wiig E., Reseland J.E., Johnsen G.F., Silva E.J., Haugen H.J. (2023). Regenerative endodontics by cell homing: A review of recent clinical trials. J. Endod..

[61] Fatehi F. (2013). Natural scaffold materials used in regenerative endodontic: A review. J. Biomater. Tissue Eng..

[62] Kim D.S., Park H.J., Yeom J.H., Seo J.S., Ryu G.J., Park K.H., Shin S.I., Kim S.Y. (2012). Long-term follow-ups of revascularized immature necrotic teeth: Three case reports. Int. J. Oral Sci..

[63] Siddiqui Z., Acevedo-Jake A.M., Griffith A., Kadincesme N., Dabek K., Hindi D., Kim K.K., Kobayashi Y., Shimizu E., Kumar V. (2022). Cells and material-based strategies for regenerative endodontics. Bioact. Mater..

[64] Nakashima M., Akamine A. (2005). The application of tissue engineering to regeneration of pulp and dentin in endodontics. J. Endod..

[65] Pozos-Guillén A., Flores H. (2020). Dentin-pulp complex regeneration. Current Advances in Oral and Craniofacial Tissue Engineering.

[66] Albuquerque M.T.P., Valera M.C., Nakashima M., Nör J.E., Bottino M.C. (2014). Tissue-engineering-based strategies for regenerative endodontics. J. Dent. Res..

[67] Alkhursani S.A., Ghobashy M.M., Al-Gahtany S.A., Meganid A.S., Abd El-Halim S.M., Ahmad Z., Khan F.S., Atia G.A.N., Cavalu S. (2022). Application of nano-inspired scaffolds-based biopolymer hydrogel for bone and periodontal tissue regeneration. Polymers.

[68] Dubey N., Ribeiro J.S., Zhang Z., Xu J., Ferreira J.A., Qu L., Mei L., Fenno J.C., Schwendeman A., Schwendeman S.P. (2023). Gelatin methacryloyl hydrogel as an injectable scaffold with multi-therapeutic effects to promote antimicrobial disinfection and angiogenesis for regenerative endodontics. J. Mater. Chem. B.

[69] Costa L.A., Eiro N., Vaca A., Vizoso F.J. (2022). Towards a new concept of regenerative endodontics based on mesenchymal stem cell-derived secretomes products. Bioengineering.

[70] Sequeira D.B., Diogo P., Gomes B.P., Peça J., Santos J.M.M. (2023). Scaffolds for Dentin–Pulp Complex Regeneration. Medicina.

[71] Orti V., Collart-Dutilleul P.Y., Piglionico S., Pall O., Cuisinier F., Panayotov I. (2018). Pulp regeneration concepts for nonvital teeth: From tissue engineering to clinical approaches. Tissue Eng. Part B Rev..

[72] Gelman R., Park H. (2012). Pulp revascularization in an immature necrotic tooth: A case report. Pediatr. Dent..

[73] Moussa D.G., Aparicio C. (2019). Present and future of tissue engineering scaffolds for dentin-pulp complex regeneration. J. Tissue Eng. Regen. Med..

[74] Ahmed H.M., Duncan H.F., El-Karim I.A., Cooper P.R. (2024). Dental Pulp Stem Cells in Endodontics: Advances, Applications, and Challenges. Handbook of Stem Cell Applications.

[75] Rahman S.U., Nagrath M., Ponnusamy S., Arany P.R. (2018). Nanoscale and macroscale scaffolds with controlled-release polymeric systems for dental craniomaxillofacial tissue engineering. Materials.

[76] Iranmanesh P., Ehsani A., Khademi A., Asefnejad A., Shahriari S., Soleimani M., Ghadiri Nejad M., Saber-Samandari S., Khandan A. (2022). Application of 3D bioprinters for dental pulp regeneration and tissue engineering (porous architecture). Transp. Porous Media.

[77] EzEldeen M., Moroni L., Nejad Z.M., Jacobs R., Mota C. (2023). Biofabrication of engineered dento-alveolar tissue. Biomater. Adv..

[78] Choi D., Qiu M., Hwang Y.C., Oh W.M., Koh J.T., Park C., Lee B.N. (2022). The Effects of 3-dimensional bioprinting calcium silicate cement/methacrylated gelatin scaffold on the proliferation and differentiation of human dental pulp stem cells. Materials.

[79] Jadhav G., Shah N., Logani A. (2012). Revascularization with and without platelet-rich plasma in nonvital, immature, anterior teeth: A pilot clinical study. J. Endod..

[80] Nosrat A., Homayounfar N., Oloomi K. (2012). Drawbacks and unfavorable outcomes of regenerative endodontic treatments of necrotic immature teeth: A literature review and report of a case. J. Endod..

[81] Anderson J., Wealleans J., Ray J. (2018). Endodontic applications of 3D printing. Int. Endod. J..

[82] Yang H., Fang H., Wang C., Wang Y., Qi C., Zhang Y., Zhou Q., Huang M., Wang M., Wu M. (2023). 3D printing of customized functional devices for smart biomedical systems. SmartMat.

[83] Kustra P., Dobroś K., Zarzecka J. (2021). Making use of three-dimensional models of teeth, manufactured by stereolithographic technology, in practical teaching of endodontics. Eur. J. Dent. Educ..

[84] Zhang T., Chen D., Zhang F., Xie S., Wu G., Hu Q., Yan F., Tang X. (2024). Comparison of selective laser melting and stereolithography etching templates for guided endodontics. PeerJ.

[85] Alageel O., Wazirian B., Almufleh B., Tamimi F. (2019). Fabrication of dental restorations using digital technologies: Techniques and materials. Digital Restorative Dentistry: A Guide to Materials, Equipment, and Clinical Procedures.

[86] Liu Y., Liang L., Rajan S.S., Damade Y., Zhang X., Mishra K., Qu L., Dubey N. (2024). Recent advances in additive manufacturing for tooth restorations. Appl. Mater. Today.

[87] Ashammakhi N., GhavamiNejad A., Tutar R., Fricker A., Roy I., Chatzistavrou X., Hoque Apu E., Nguyen K.L., Ahsan T., Pountos I. (2022). Highlights on advancing frontiers in tissue engineering. Tissue Eng. Part B Rev..

[88] Umapathy V.R., Natarajan P.M., Swamikannu B. (2025). Regenerative Strategies in Dentistry: Harnessing Stem Cells, Biomaterials and Bioactive Materials for Tissue Repair. Biomolecules.

[89] Gong T., Heng B.C., Lo E.C.M., Zhang C. (2016). Current advance and future prospects of tissue engineering approach to dentin/pulp regenerative therapy. Stem Cells Int..

[90] Makandar S.D. (2021). Regenerative Endodontics-challenges and Future Direction. Int. J. Innov. Eng. Res. Technol..

[91] Chavez-Granados P.A., Manisekaran R., Acosta-Torres L.S., Garcia-Contreras R. (2022). CRISPR/Cas gene-editing technology and its advances in dentistry. Biochimie.

[92] Marvaniya J., Agarwal K., Mehta D.N., Parmar N., Shyamal R., Patel J. (2022). Minimal invasive endodontics: A comprehensive narrative review. Cureus.

[93] Fu X., Kim H.S. (2024). Dentin Mechanobiology: Bridging the Gap between Architecture and Function. Int. J. Mol. Sci..

[94] Malhotra N. (2019). Bioreactors design, types, influencing factors and potential application in dentistry. A literature review. Curr. Stem Cell Res. Ther..

[95] Iviglia G., Kargozar S., Baino F. (2019). Biomaterials, current strategies, and novel nano-technological approaches for periodontal regeneration. J. Funct. Biomater..

[96] Wang Y., Zhuo L., Hu X., Lu S., Dong C. (2024). Unveiling the future of endodontics: An update on dental pulp regeneration strategies. Saudi Dent. J..

[97] Alyafei S.H., Anil S. (2024). Regenerative Approaches in Gingival Tissue Engineering. Advances in Gingival Diseases and Conditions.

[98] Takeuchi N., Hayashi Y., Murakami M., Alvarez F.J., Horibe H., Iohara K., Nakata K., Nakamura H., Nakashima M. (2015). Similar in vitro effects and pulp regeneration in ectopic tooth transplantation by basic fibroblast growth factor and granulocyte-colony stimulating factor. Oral Dis..

[99] de Pablo J.A., Serrano L.J., García-Arranz M., Romeu L., Liras A. (2021). Gene and Cell Therapy in Dental Tissue Regeneration. Human Tooth and Developmental Dental Defects—Compositional and Genetic Implications.

[100] Orduña J.F.G., Caviedes-Bucheli J., Céspedes M.C.M., Jimeno E.B., Biedma B.M., Segura-Egea J.J., López-López J. (2017). Use of platelet-rich plasma in endodontic procedures in adults: Regeneration or repair? A report of 3 cases with 5 years of follow-up. J. Endod..

[101] Cabaña-Muñoz M.E., Pelaz Fernández M.J., Parmigiani-Cabaña J.M., Parmigiani-Izquierdo J.M., Merino J.J. (2023). Adult mesenchymal stem cells from oral cavity and surrounding areas: Types and biomedical applications. Pharmaceutics.

[102] Hashemi-Beni B., Khoroushi M., Foroughi M.R., Karbasi S., Khademi A.A. (2017). Tissue engineering: Dentin–pulp complex regeneration approaches (A review). Tissue Cell.

[103] Kwack K.H., Lee H.W. (2022). Clinical potential of dental pulp stem cells in pulp regeneration: Current endodontic progress and future perspectives. Front. Cell Dev. Biol..

[104] Chen X.D., Chen X.D. (2018). A Roadmap to Nonhematopoietic Stem Cell-Based Therapeutics: From the Bench to the Clinic.

[105] Eramo S., Natali A., Pinna R., Milia E. (2018). Dental pulp regeneration via cell homing. Int. Endod. J..

[106] Egusa H., Sonoyama W., Nishimura M., Atsuta I., Akiyama K. (2012). Stem cells in dentistry–Part II: Clinical applications. J. Prosthodont. Res..

[107] Huang G.J., Gronthos S., Shi S. (2009). Mesenchymal stem cells derived from dental tissues vs. those from other sources: Their biology and role in regenerative medicine. J. Dent. Res..

[108] Collart-Dutilleul P.Y., Chaubron F., De Vos J., Cuisinier F.J. (2015). Allogenic banking of dental pulp stem cells for innovative therapeutics. World J. Stem Cells.

[109] Wang L.H., Gao S.Z., Bai X.L., Chen Z.L., Yang F. (2022). An up-to-date overview of dental tissue regeneration using dental origin mesenchymal stem cells: Challenges and road ahead. Front. Bioeng. Biotechnol..

[110] Botelho J., Cavacas M.A., Machado V., Mendes J.J. (2017). Dental stem cells: Recent progresses in tissue engineering and regenerative medicine. Ann. Med..

[111] Goldberg M. (2017). Stem cells: Tools for dental tissues regeneration. J. Dent. Health Oral Disord Ther..

[112] Zhang W., Yelick P.C. (2021). Tooth repair and regeneration: Potential of dental stem cells. Trends Mol. Med..

[113] Deepyanti D., Srishti D., Ghosh D.M., Saha D.A. (2021). Regenerative Endodontics.

[114] Rosa V. (2013). What and where are the stem cells for dentistry?. Singap. Dent. J..

[115] Bansal R., Bansal R. (2011). Regenerative endodontics: A state of the art. Indian J. Dent. Res..

[116] Zakrzewski W., Dobrzyński M., Szymonowicz M., Rybak Z. (2019). Stem cells: Past, present, and future. Stem Cell Res. Ther..

[117] Cao Y., Song M., Kim E., Shon W., Chugal N., Bogen G., Lin L., Kim R.H., Park N.H., Kang M.K. (2015). Pulp-dentin regeneration: Current state and future prospects. J. Dent. Res..

[118] KBatsali A., Kastrinaki M.C., APapadaki H., Pontikoglou C. (2013). Mesenchymal stem cells derived from Wharton’s Jelly of the umbilical cord: Biological properties and emerging clinical applications. Curr. Stem Cell Res. Ther..

[119] Xie Z., Shen Z., Zhan P., Yang J., Huang Q., Huang S., Chen L., Lin Z. (2021). Functional dental pulp regeneration: Basic research and clinical translation. Int. J. Mol. Sci..

[120] Shaikh M.S., Shahzad Z., Tash E.A., Janjua O.S., Khan M.I., Zafar M.S. (2022). Human umbilical cord mesenchymal stem cells: Current literature and role in periodontal regeneration. Cells.

[121] Nada O.A., El Backly R.M. (2018). Stem cells from the apical papilla (SCAP) as a tool for endogenous tissue regeneration. Front. Bioeng. Biotechnol..

[122] Nakashima M., Iohara K., Murakami M. (2013). Dental pulp stem cells and regeneration. Endod. Top..

[123] Galler K.M., Weber M., Korkmaz Y., Widbiller M., Feuerer M. (2021). Inflammatory response mechanisms of the dentine–pulp complex and the periapical tissues. Int. J. Mol. Sci..

[124] Hernández-Monjaraz B., Santiago-Osorio E., Monroy-García A., Ledesma-Martínez E., Mendoza-Núñez V.M. (2018). Mesenchymal stem cells of dental origin for inducing tissue regeneration in periodontitis: A mini-review. Int. J. Mol. Sci..

[125] Nikoloudaki G. (2021). Functions of matricellular proteins in dental tissues and their emerging roles in orofacial tissue development, maintenance, and disease. Int. J. Mol. Sci..

[126] Zhang F., Song J., Zhang H., Huang E., Song D., Tollemar V., Wang J., Wang J., Mohammed M., Wei Q. (2016). Wnt and BMP signaling crosstalk in regulating dental stem cells: Implications in dental tissue engineering. Genes Dis..

[127] Zhang S., Zhang W., Li Y., Ren L., Deng H., Yin X., Gao X., Pan S., Niu Y. (2020). Human umbilical cord mesenchymal stem cell differentiation into odontoblast-like cells and endothelial cells: A potential cell source for dental pulp tissue engineering. Front. Physiol..

[128] Kiarashi M., Bayat H., Shahrtash S.A., Etajuri E.A., Khah M.M., Al-Shaheri N.A., Nasiri K., Esfahaniani M., Yasamineh S. (2024). Mesenchymal stem cell-based scaffolds in regenerative medicine of dental diseases. Stem Cell Rev. Rep..

[129] Mantesso A., Sharpe P. (2009). Dental stem cells for tooth regeneration and repair. Expert Opin. Biol. Ther..

[130] Smith A.J., Duncan H.F., Diogenes A., Simon S., Cooper P.R. (2016). Exploiting the bioactive properties of the dentin-pulp complex in regenerative endodontics. J. Endod..

[131] Donnaloja F., Jacchetti E., Soncini M., Raimondi M.T. (2020). Natural and synthetic polymers for bone scaffolds optimization. Polymers.

[132] Watcharadulyarat N., Rattanatayarom M., Ruangsawasdi N., Hongdilokkul N., Patikarnmonthon N. (2022). Dextran-Based Nanoparticles for Encapsulation of Ciprofloxacin. Proceedings of the 21st International Union of Materials Research Societies.

[133] Paul M., Pramanik S.D., Sahoo R.N., Dey Y.N., Nayak A.K. (2023). Dental delivery systems of antimicrobial drugs using chitosan, alginate, dextran, cellulose and other polysaccharides: A review. Int. J. Biol. Macromol..

[134] Murad A.H. (2023). Immunomodulatory Biomaterials in Dental Pulp Regenera-tion: Towards Enhancing Endodontic Treatment Outcomes. Future Dent. Res..

[135] Marei M. (2010). Regenerative Dentistry.

[136] Rad R.M., Atila D., Akgün E.E., Evis Z., Keskin D., Tezcaner A. (2019). Evaluation of human dental pulp stem cells behavior on a novel nanobiocomposite scaffold prepared for regenerative endodontics. Mater. Sci. Eng. C.

[137] Rohban R., Pieber T.R. (2017). Mesenchymal stem and progenitor cells in regeneration: Tissue specificity and regenerative potential. Stem Cells Int..

[138] Al-Sharabi N., Xue Y., Fujio M., Ueda M., Gjerde C., Mustafa K., Fristad I. (2014). Bone marrow stromal cell paracrine factors direct osteo/odontogenic differentiation of dental pulp cells. Tissue Eng. Part A.

[139] Noohi P., Abdekhodaie M.J., Nekoofar M.H., Galler K.M., Dummer P.M. (2022). Advances in scaffolds used for pulp–dentine complex tissue engineering: A narrative review. Int. Endod. J..

[140] Farjaminejad S., Shojaei S., Goodarzi V., Khonakdar H.A., Abdouss M. (2021). Tuning properties of bio-rubbers and its nanocomposites with addition of succinic acid and ε-caprolactone monomers to poly (glycerol sebacic acid) as main platform for application in tissue engineering. Eur. Polym. J..

[141] Huang X., Li Z., Liu A., Liu X., Guo H., Wu M., Yang X., Han B., Xuan K. (2021). Microenvironment influences odontogenic mesenchymal stem cells mediated dental pulp regeneration. Front. Physiol..

[142] Williams A.G., Moore E., Thomas A., Johnson J.A. (2023). Graphene-Based Materials in Dental Applications: Antibacterial, Biocompatible, and Bone Regenerative Properties. Int. J. Biomater..

[143] Mkhabela V.J. (2015). Novel Bio-Nanocomposite Scaffolds for Tissue Engineering Application. Ph.D. Thesis.

[144] Kim D.S., Kim Y.S., Bae W.J., Lee H.J., Chang S.W., Kim W.S., Kim E.C. (2014). The role of SDF-1 and CXCR 4 on odontoblastic differentiation in human dental pulp cells. Int. Endod. J..

[145] Farjaminejad S., Farjaminejad R., Hasani M., Garcia-Godoy F., Abdouss M., Marya A., Harsoputranto A., Jamilian A. (2024). Advances and Challenges in Polymer-Based Scaffolds for Bone Tissue Engineering: A Path Towards Personalized Regenerative Medicine. Polymers.

[146] Riaz A., Shah F.A. (2021). Regenerating the pulp–dentine complex using autologous platelet concentrates: A critical appraisal of the current histological evidence. Tissue Eng. Regen. Med..

[147] Wang L., Zhao Y., Shi S. (2012). Interplay between mesenchymal stem cells and lymphocytes: Implications for immunotherapy and tissue regeneration. J. Dent. Res..

[148] Ulusoy A.T., Turedi I., Cimen M., Cehreli Z.C. (2019). Evaluation of blood clot, platelet-rich plasma, platelet-rich fibrin, and platelet pellet as scaffolds in regenerative endodontic treatment: A prospective randomized trial. J. Endod..

[149] Bakhtiar H., Esmaeili S., Tabatabayi S.F., Ellini M.R., Nekoofar M.H., Dummer P.M. (2017). Second-generation platelet concentrate (platelet-rich fibrin) as a scaffold in regenerative endodontics: A case series. J. Endod..

[150] Song J.S., Takimoto K., Jeon M., Vadakekalam J., Ruparel N.B., Diogenes A. (2017). Decellularized human dental pulp as a scaffold for regenerative endodontics. J. Dent. Res..

[151] Bakhtiar H., Rajabi S., Pezeshki-Modaress M., Ellini M.R., Panahinia M., Alijani S., Mazidi A., Kamali A., Azarpazhooh A., Kishen A. (2021). Optimizing methods for bovine dental pulp decellularization. J. Endod..

[152] Sumita Y., Honda M.J., Ohara T., Tsuchiya S., Sagara H., Kagami H., Ueda M. (2006). Performance of collagen sponge as a 3-D scaffold for tooth-tissue engineering. Biomaterials.

[153] Jiang X., Liu H., Peng C. (2017). Clinical and radiographic assessment of the efficacy of a collagen membrane in regenerative endodontics: A randomized, controlled clinical trial. J. Endod..

[154] Ahsan S.M., Thomas M., Reddy K.K., Sooraparaju S.G., Asthana A., Bhatnagar I. (2018). Chitosan as biomaterial in drug delivery and tissue engineering. Int. J. Biol. Macromol..

[155] Bakopoulou A., Georgopoulou A., Grivas I., Bekiari C., Prymak O., Loza Κ., Epple M., Papadopoulos G.C., Koidis P., Chatzinikolaidou Μ. (2019). Dental pulp stem cells in chitosan/gelatin scaffolds for enhanced orofacial bone regeneration. Dent. Mater..

[156] Lee K.Y., Mooney D.J. (2012). Alginate: Properties and biomedical applications. Prog. Polym. Sci..

[157] Lambricht L., De Berdt P., Vanacker J., Leprince J., Diogenes A., Goldansaz H., Bouzin C., Préat V., Dupont-Gillain C., Des Rieux A. (2014). The type and composition of alginate and hyaluronic-based hydrogels influence the viability of stem cells of the apical papilla. Dent. Mater..

[158] Virlan M.J.R., Calenic B., Zaharia C., Greabu M. (2015). Silk fibroin and potential uses in regenerative dentistry—A systematic review. Stomatol. Edu J..

[159] Narayanam R., Cardoso L.M., dos Reis-Prado A.H., de Carvalho A.B.G., Anselmi C., Mahmoud A.H., Fenno J.C., Dal-Fabbro R., Bottino M.C. (2024). Antimicrobial Silk Fibroin Methacrylated Scaffolds for Regenerative Endodontics. J. Endod..

[160] AlHowaish N.A., AlSudani D.I., AlMuraikhi N.A. (2022). Evaluation of a hyaluronic acid hydrogel (Restylane Lyft) as a scaffold for dental pulp regeneration in a regenerative endodontic organotype model. Odontology.

[161] Casale M., Moffa A., Vella P., Sabatino L., Capuano F., Salvinelli B., Lopez M.A., Carinci F., Salvinelli F. (2016). Hyaluronic acid: Perspectives in dentistry. A systematic review. Int. J. Immunopathol. Pharmacol..

[162] Caballero-Flores H., Nabeshima C.K., Sarra G., Moreira M.S., Arana-Chavez V.E., Marques M.M., de Lima Machado M.E. (2021). Development and characterization of a new chitosan-based scaffold associated with gelatin, microparticulate dentin and genipin for endodontic regeneration. Dent. Mater..

[163] Ribeiro J.S., Sanz C.K., Münchow E.A., Kalra N., Dubey N., Suárez C.E.C., Fenno J.C., Lund R.G., Bottino M.C. (2022). Photocrosslinkable methacrylated gelatin hydrogel as a cell-friendly injectable delivery system for chlorhexidine in regenerative endodontics. Dent. Mater..

[164] Alghofaily M., Almana A., Alrayes J., Lambarte R., Weir M.D., Alsalleeh F. (2024). Chitosan–Gelatin Scaffolds Loaded with Different Antibiotic Formulations for Regenerative Endodontic Procedures Promote Biocompatibility and Antibacterial Activity. J. Funct. Biomater..

[165] Heggendorn F.L., Nascimento M.B.D., Lima A.M., Ribeiro A.A. (2023). Demineralized dentin matrix technique-a comparison of different demineralizing solutions. Braz. Dent. J..

[166] Gao X., Qin W., Wang P., Wang L., Weir M.D., Reynolds M.A., Zhao L., Lin Z., Xu H.H. (2019). Nano-structured demineralized human dentin matrix to enhance bone and dental repair and regeneration. Appl. Sci..

[167] Sanz J.L., Rodriguez-Lozano F.J., Lopez-Gines C., Monleon D., Llena C., Forner L. (2021). Dental stem cell signaling pathway activation in response to hydraulic calcium silicate-based endodontic cements: A systematic review of in vitro studies. Dent. Mater..

[168] Camilleri J., Atmeh A., Li X., Meschi N. (2022). Present status and future directions: Hydraulic materials for endodontic use. Int. Endod. J..

[169] Demirkaya K., Can Demirdöğen B., Öncel Torun Z., Erdem O., Tunca Y.M. (2018). The effects of hydraulic calcium silicate containing endodontic materials on oxidative stress in erythrocytes and liver. Turk. J. Biochem..

[170] Dobrzańska J., Gołombek K., Dobrzański L.B. (2012). Polymer materials used in endodontic treatment-in vitro testing. Arch. Mater. Sci. Eng..

[171] Zein N., Harmouch E., Lutz J.C., Fernandez De Grado G., Kuchler-Bopp S., Clauss F., Offner D., Hua G., Benkirane-Jessel N., Fioretti F. (2019). Polymer-based instructive scaffolds for endodontic regeneration. Materials.

[172] Aksel H., Mahjour F., Bosaid F., Calamak S., Azim A.A. (2020). Antimicrobial activity and biocompatibility of antibiotic-loaded chitosan hydrogels as a potential scaffold in regenerative endodontic treatment. J. Endod..

[173] Duncan H.F., Cooper P.R. (2022). The bioactive properties of dentine and molecular advances in pulp regeneration. Endodontic Advances and Evidence-Based Clinical Guidelines.

[174] Brizuela C., Huang G.T.J., Diogenes A., Botero T., Khoury M. (2022). The four pillars for successful regenerative therapy in endodontics: Stem cells, biomaterials, growth factors, and their synergistic interactions. Stem Cells Int..

[175] Zeng Q., Nguyen S., Zhang H., Chebrolu H.P., Alzebdeh D., Badi M.A., Kim J.R., Ling J., Yang M. (2016). Release of growth factors into root canal by irrigations in regenerative endodontics. J. Endod..

[176] Mandracci P., Mussano F., Rivolo P., Carossa S. (2016). Surface treatments and functional coatings for biocompatibility improvement and bacterial adhesion reduction in dental implantology. Coatings.

[177] Nakashima M. (2005). Bone morphogenetic proteins in dentin regeneration for potential use in endodontic therapy. Cytokine Growth Factor Rev..

[178] Huang G.J., Garcia-Godoy F. (2014). Missing concepts in de novo pulp regeneration. J. Dent. Res..

[179] Cassidy N., Fahey M., Prime S.S., Smith A.J. (1997). Comparative analysis of transforming growth factor-beta isoforms 1-3 in human and rabbit dentine matrices. Arch. Oral Biol..

[180] Galler K.M., Buchalla W., Hiller K.A., Federlin M., Eidt A., Schiefersteiner M., Schmalz G. (2015). Influence of root canal disinfectants on growth factor release from dentin. J. Endod..

[181] Smith A.J., Lesot H. (2001). Induction and regulation of crown dentinogenesis: Embryonic events as a template for dental tissue repair?. Crit. Rev. Oral Biol. Med..

[182] Thesleff I., Vaahtokari A., Partanen A.M. (1995). Regulation of organogenesis: Common molecular mechanisms regulating the development of teeth and other organs. Int. J. Dev. Biol..

[183] Liu J., Jin T., Chang S., Ritchie H.H., Smith A.J., Clarkson B.H. (2007). Matrix and TGF-beta-related gene expression during human dental pulp stem cell (DPSC) mineralization. Vitr. Cell. Dev. Biol.-Anim..

[184] Huojia M., Muraoka N., Yoshizaki K., Fukumoto S., Nakashima M., Akamine A., Nonaka K., Ohishi M. (2005). TGF-beta3 induces ectopic mineralization in fetal mouse dental pulp during tooth germ development. Dev. Growth Differ..

[185] Sloan A.J., Smith A.J. (1999). Stimulation of the dentine-pulp complex of rat incisor teeth by transforming growth factor-beta isoforms 1–3 in vitro. Arch. Oral Biol..

[186] Thomadakis G., Ramoshebi L.N., Crooks J., Rueger D.C., Ripamonti U. (1999). Immunolocalization of bone morphogenetic protein-2 and -3 and osteogenic protein-1 during murine tooth root morphogenesis and in other craniofacial structures. Eur. J. Oral Sci..

[187] Iohara K., Nakashima M., Ito M., Ishikawa M., Nakasima A., Akamine A. (2004). Dentin regeneration by dental pulp stem cell therapy with recombinant human bone morphogenetic protein 2. J. Dent. Res..

[188] Chen S., Gluhak-Heinrich J., Martinez M., Li T., Wu Y., Chuang H.H., Chen L., Dong J., Gay I., MacDougall M. (2008). Bone morphogenetic protein 2 mediates dentin sialophosphoprotein expression and odontoblast differentiation via NF-Y signaling. J. Biol. Chem..

[189] About I., Laurent-Maquin D., Lendahl U., Mitsiadis T.A. (2000). Nestin expression in embryonic and adult human teeth under normal and pathological conditions. Am. J. Pathol..

[190] Helder M.N., Karg H., Bervoets T.J.M., Vukicevic S., Burger E.H., D’souza R.N., Wöltgens J.H.M., Karsenty G., Bronckers A.L.J.J. (1998). Bone morphogenetic protein-7 (osteogenic protein-1, OP-1) and tooth development. J. Dent. Res..

[191] Suzuki T., Lee C.H., Chen M., Zhao W., Fu S.Y., Qi J.J., Chotkowski G., Eisig S.B., Wong A., Mao J.J. (2011). Induced migration of dental pulp stem cells for in vivo pulp regeneration. J. Dent. Res..

[192] Kim K., Lee C.H., Kim B.K., Mao J.J. (2010). Anatomically shaped tooth and periodontal regeneration by cell homing. J. Dent. Res..

[193] Finkelman R.D., Mohan S., Jennings J.C., Taylor A.K., Jepsen S., Baylink D.J. (1990). Quantitation of growth factors IGF-I, SGF/IGF-II, and TGF-beta in human dentin. J. Bone Miner. Res..

[194] Duncan H.F., Smith A.J., Fleming G.J.P., Reid C., Smith G., Cooper P.R. (2015). Release of bio-active dentine extracellular matrix components by histone deacetylase inhibitors (HDACi). Int. Endod. J..

[195] Feng X., Huang D., Lu X., Feng G., Xing J., Lu J., Xu K., Xia W., Meng Y., Tao T. (2014). Insulin-like growth factor 1 can promote proliferation and osteogenic differentiation of human dental pulp stem cells via mTOR pathway. Dev. Growth Differ..

[196] Wang S., Mu J., Fan Z., Yu Y., Yan M., Lei G., Tang C., Wang Z., Zheng Y., Yu J. (2012). Insulin-like growth factor 1 can promote the osteogenic differentiation and osteogenesis of stem cells from apical papilla. Stem Cell Res..

[197] Tomson P.L., Lumley P.J., Alexander M.Y., Smith A.J., Cooper P.R. (2013). Hepatocyte growth factor is sequestered in dentine matrix and promotes regeneration-associated events in dental pulp cells. Cytokine.

[198] Forte G., Minieri M., Cossa P., Antenucci D., Sala M., Gnocchi V., Fiaccavento R., Carotenuto F., De Vito P., Baldini P.M. (2006). Hepatocyte growth factor effects on mesenchymal stem cells: Proliferation, migration, and differentiation. Stem Cells.

[199] Roberts-Clark D.J., Smith A.J. (2000). Angiogenic growth factors in human dentine matrix. Arch. Oral Biol..

[200] Musson D.S., McLachlan J.L., Sloan A.J., Smith A.J., Cooper P.R. (2010). Adrenomedullin is expressed during rodent dental tissue development and promotes cell growth and mineralization. Biol. Cell.

[201] Tomson P.L., Grover L.M., Lumley P.J., Sloan A.J., Smith A.J., Cooper P.R. (2007). Dissolution of bio-active dentine matrix components by mineral trioxide aggregate. J. Dent..

[202] Simon S., Smith A.J., Berdal A., Lumley P.J., Cooper P.R. (2010). The MAP kinase pathway is involved in odontoblast stimulation via p38 phosphorylation. J. Endod..

[203] Morelli T., Neiva R., Nevins M.L., McGuire M.K., Scheyer E.T., Oh T.J., Braun T.M., Nör J.E., Bates D., Giannobile W.V. (2011). Angiogenic biomarkers and healing of living cellular constructs. J. Dent. Res..

[204] Fiedler J., Roderer G., Gunther K.P., Brenner R.E. (2002). BMP-2, BMP-4, and PDGF-bb stimulate chemotactic migration of primary human mesenchymal progenitor cells. J. Cell Biochem..

[205] Yokose S., Kadokura H., Tajima N., Hasegawa A., Sakagami H., Fujieda K., Katayama T. (2004). Platelet-derived growth factor exerts disparate effects on odontoblast differentiation depending on the dimers in rat dental pulp cells. Cell Tissue Res..

[206] Kim J.Y., Xin X., Moioli E.K., Chung J., Lee C.H., Chen M., Fu S.Y., Koch P.D., Mao J.J. (2010). Regeneration of dental-pulp-like tissue by chemotaxis-induced cell homing. Tissue Eng. Part A.

[207] Arthur A., Rychkov G., Shi S., Koblar S.A., Gronthos S. (2008). Adult human dental pulp stem cells differentiate toward functionally active neurons under appropriate environmental cues. Stem Cells.

[208] Vanacker J., Viswanath A., De Berdt P., Everard A., Cani P.D., Bouzin C., Feron O., Diogenes A., Leprince J.G., des Rieux A. (2014). Hypoxia modulates the differentiation potential of stem cells of the apical papilla. J. Endod..

[209] Kinnaird T., Stabile E., Burnett M.S., Shou M., Lee C.W., Barr S., Fuchs S., Epstein S.E. (2004). Local delivery of marrow-derived stromal cells augments collateral perfusion through paracrine mechanisms. Circulation.

[210] McCoy R.J., Widaa A., Watters K.M., Wuerstle M., Stallings R.L., Duffy G.P., O’Brien F.J. (2013). Orchestrating osteogenic differentiation of mesenchymal stem cells: Identification of placental growth factor as a mechano sensitive gene with a pro-osteogenic role. Stem Cells.

[211] de Almeida J.F., Chen P., Henry M.A., Diogenes A. (2014). Stem cells of the apical papilla regulate trigeminal neurite outgrowth and targeting through a BDNF-dependent mechanism. Tissue Eng. Part A.

[212] Marquardt L.M., Ee X., Iyer N., Hunter D., Mackinnon S.E., Wood M.D., Sakiyama-Elbert S.E. (2015). Finely tuned temporal and spatial delivery of GDNF promotes enhanced nerve regeneration in a long nerve defect model. Tissue Eng. Part A.

[213] Gale Z., Cooper P.R., Scheven B.A. (2011). Effects of glial cell line-derived neurotrophic factor on dental pulp cells. J. Dent. Res..

[214] Müller A.S., Artner M., Janjić K., Edelmayer M., Kurzmann C., Moritz A., Agis H. (2018). Synthetic Clay–based Hypoxia Mimetic Hydrogel for Pulp Regeneration: The Impact on Cell Activity and Release Kinetics Based on Dental Pulp–derived Cells In Vitro. J. Endod..

[215] Zhang R., Xie L., Wu H., Yang T., Zhang Q., Tian Y., Liu Y., Han X., Guo W., He M. (2020). Alginate/laponite hydrogel microspheres co-encapsulating dental pulp stem cells and VEGF for endodontic regeneration. Acta Biomater..

[216] Wei Y., Lyu P., Bi R., Chen X., Yu Y., Li Z., Fan Y. (2022). Neural regeneration in regenerative endodontic treatment: An overview and current trends. Int. J. Mol. Sci..

